# Beyond binary classification: Comparing three region‐based multi‐phase Aβ staging systems

**DOI:** 10.1002/alz.70253

**Published:** 2025-05-19

**Authors:** Liang Cui, Zhen Zhang, Chu‐Chung Huang, Cheng‐Yi Yuan, You‐Yi Tu, Min Wang, Yi‐Hui Guan, Yue‐Hua Li, Fang Xie, Qi‐Hao Guo

**Affiliations:** ^1^ Department of Gerontology Shanghai Sixth People's Hospital Affiliated to Shanghai Jiao Tong University School of Medicine Shanghai China; ^2^ Shanghai Key Laboratory of Brain Functional Genomics (Ministry of Education) Affiliated Mental Health Center (ECNU) School of Psychology and Cognitive Science East China Normal University Shanghai China; ^3^ Shanghai Changning Mental Health Center Shanghai China; ^4^ School of Life Sciences Shanghai University Shanghai China; ^5^ Department of Nuclear Medicine & PET Center Huashan Hospital Fudan University Shanghai China; ^6^ Department of Radiology Shanghai Sixth People's Hospital Affiliated to Shanghai Jiao Tong University School of Medicine Shanghai China

**Keywords:** Alzheimer's disease, amyloid‐beta, cognitive function, default mode network, deposition stage, hippocampus, neurodegeneration, plasma biomarkers

## Abstract

**INTRODUCTION:**

This study aims to establish a multi‐phase visual staging system based on amyloid‐beta (Aβ) deposition to more precisely assess the stages of Aβ accumulation in Alzheimer's disease (AD), and to validate the cognitive function, neurodegeneration, and blood biomarker characteristics at different stages.

**METHODS:**

A total of 1002 participants from the Chinese Preclinical Alzheimer's Disease Study (C‐PAS) cohort were included. Aβ deposition was assessed using positron emission tomography (PET) imaging, and three multi‐phase Aβ deposition grading systems were established. Participants underwent neuropsychological testing, peripheral blood biomarker collection, and multimodal neuroimaging data acquisition.

**RESULTS:**

Cognitive function, peripheral blood biomarkers, default mode network (DMN) function, and hippocampal volumes showed stage‐dependent changes across different Aβ deposition stages. Different grading systems revealed varying clinical manifestations and biomarker sensitivity.

**DISCUSSION:**

Region‐based multi‐phase Aβ deposition systems demonstrate practical utility in detecting early pathological changes, understanding disease progression, and informing early diagnosis and intervention strategies for AD.

**Highlights:**

- Developed and validated visual multi‐phase Aβ deposition staging systems for AD progression.
- Revealed stage‐specific cognitive, neurodegenerative, and biomarker characteristics in AD.
- Demonstrated the sensitivity of visual methods to detect early, regional Aβ deposition.
- Highlighted differential strengths of Villeneuve, Grothe, and Mattsson staging systems.
- Proposed multi‐phase systems as tools for personalized AD diagnosis and intervention strategies.

## 1 INTRODUCTION

Alzheimer's disease (AD) is a neurodegenerative disorder characterized by the progressive decline in cognitive function, which is closely associated with the accumulation of amyloid‐beta (Aβ) plaques in the brain.^1^ Positron emission tomography (PET) is widely used for non‐invasive detection of Aβ deposition, often interpreted through binary classification as positive or negative. However, this approach creates a “gray zone,”^2^, ^3^ particularly in preclinical or early disease stages,^4^ hindering accurate assessment. Quantitative methods like standardized uptake value ratio (SUVR) or centiloid measure overall Aβ levels but may miss region‐specific early deposition. Visual assessment, while capable of detecting focal signals for early staging,^5^, ^6^ lacks a standardized system for evaluating pathology severity.

Some studies have shown that Aβ deposition does not occur synchronously across different brain regions but follows a specific spatial sequence.^7^, ^8^, ^9^ This spatial sequence provides a theoretical basis for staging the progression of Aβ deposition. Research has confirmed that region‐based Aβ‐PET staging methods outperform standard global Aβ‐PET signal analysis in distinguishing neuropathological stages in autopsy cohorts.^10^ Algorithms based on spatial information of Aβ deposition have already shown the potential to improve diagnostic accuracy.^11^ However, further work is needed to apply them in clinical practice and to explore their correlation with other pathological changes. In our previous research, we have found that regional Aβ deposition features carry important pathophysiological implications.^12^ Therefore, constructing a more refined staging system based on Aβ reginal deposition can reveal the pathological and physiological characteristics at different stages, providing strong support for a deeper understanding of the mechanisms underlying the development of AD.

Establishing multi‐phase Aβ deposition staging systems and applying them in clinical practice is of great significance for monitoring the progression of AD, achieving early accurate diagnosis, and assessing prognosis risk. These staging systems not only help identify individuals in the gray zone but also provide important support for personalized medicine. Based on the existing theory of Aβ deposition sequence, we have preliminarily constructed three multi‐phase Aβ deposition staging frameworks. Using multi‐modal neuroimaging, blood biomarkers, and neuropsychological testing data, we have detailed the clinical features of Aβ deposition at different stages. We believe that these multi‐phase, clinically operable staging systems will provide more accurate support for the early diagnosis and intervention of AD.

## 2 METHODS

### 2.1 Study design and participants

This study was an observational study conducted using data from the Chinese Preclinical Alzheimer's Disease Study (C‐PAS) cohort.^13^ The study included 1002 participants recruited between March 2019 and August 2024 from memory clinics and the community. Neuropsychological assessment, history review, and physical examination were used to determine eligibility. Participants who are native Chinese speakers, do not have severe hearing or visual impairments, and are able to complete neuropsychological testing were included in the study. The exclusion criteria are as follows: (1) individuals with a history of alcoholism or drug abuse; (2) individuals with mental illnesses, epilepsy, head trauma, stroke, or other severe neurological diseases; (3) individuals with cognitive impairments unrelated to AD (such as Parkinson's disease, Lewy body dementia, and frontotemporal dementia); 4) individuals with significant thyroid dysfunction or serological abnormalities related to syphilis.

### 2.2 Variables and outcomes

Neuropsychological testing, apolipoprotein E (APOE) genotyping, Aβ‐PET, and magnetic resonance imaging (MRI) scans were applied to all participants. Some participants underwent tau‐PET and testing for peripheral blood AD biomarkers, including plasma Aβ42, Aβ40, total‐tau (T‐tau), phosphorylated‐tau181 (P‐tau181), and neurofilament light chain (NfL).

### 2.3 Neuropsychological testing

Participants underwent the following neuropsychological tests, which included general cognition and function assessments: the Montreal Cognitive Assessment‐basic (MoCA‐B),^14^ Addenbrooke's Cognitive Examination III (ACE III),^15^ Subjective Cognitive Decline Interview (SCD‐I),^16^ Functional Activities Questionnaire (FAQ), activities of daily living (ADL); memory function assessment: Auditory Verbal Learning Test (AVLT),^17^ Brief Visuospatial Memory Test (BVMT)^18^; language function assessment: Animal‐Verbal Fluency Test (AFT), Boston Naming Test (BNT); attention and executive function assessment: Shape Trail Test (STT)‐A and STT‐B,^19^ Category Switching Test (CaST),^20^ Stroop Word‐Color Interference Task (SCWT), spatial function assessment: Silhouettes Test (ST),^21^ Judgement of Line Orientation (JLO).

### 2.4 Diagnostic criteria for cognitive impairment

Subjective cognitive decline (SCD) participants were identified based on SCD‐I criteria, requiring subjective cognitive complaints without objective impairments. Mild cognitive impairment (MCI) was diagnosed per Petersen's criteria, including objective cognitive decline but preserved daily function. Dementia was classified using the National Institute on Aging and Alzheimer's Association (NIA‐AA) criteria for probable AD. Detailed diagnostic criteria descriptions are provided in Supplementary Methods.

### 2.5 Aβ‐PET and Tau‐PET procedures

Cerebral Aβ deposition was assessed using 18F‐florbetapir PET, while tau pathology was visualized with 18F‐MK6240 PET. Both imaging protocols included standardized tracer administration, image acquisition, and reconstruction procedures. Aβ‐PET data were further processed to calculate SUVR values, using the cerebellum as the reference region. See details in Supplementary Methods.

### 2.6 Aβ‐PET and Tau‐PET visual assessment

The visual assessment of PET/CT images was performed by two trained nuclear medicine physicians who were blinded to the clinical data. In cases where there was a disagreement between the two physicians regarding the interpretation of the image results, a third physician was consulted for evaluation. The consensus reached by the two physicians served as the final result. In the judgment of Aβ‐PET results, this study employed a commonly used binary classification (negative/positive) and a region‐based multi‐phase classification method.

**RESEARCH IN CONTEXT**

1. **Systematic review**: We conducted a comprehensive review of the literature using PubMed, Scopus, and Google Scholar to identify studies focusing on amyloid‐beta (Aβ) deposition staging, neuropsychological testing, and biomarkers in Alzheimer's disease (AD). Studies on visual assessment methods, region‐based staging systems, and biomarkers (plasma Aβ, tau, and neurofilament light chain [NfL]) were prioritized. Previous research has confirmed that Aβ deposition follows a progressive, spatially distributed, multi‐phase pattern, primarily derived from quantitative, data‐driven approaches. However, there has been limited exploration of integrating these staging frameworks with multi‐modal biomarkers and cognitive assessments. Relevant references are appropriately cited.
2. **Interpretation**: Our findings validate three multi‐phase Aβ deposition systems for visual assessment, demonstrating their utility in capturing Aβ progression and stratifying its severity. These systems revealed distinct patterns of cognitive decline, hippocampal atrophy, and default mode network (DMN) functional changes at different stages. The results underscore the potential of these systems to bridge the gap between imaging‐based assessments and clinical applications, particularly in detecting early pathological changes.
3. **Future directions**: Future research should adopt longitudinal designs to assess the temporal dynamics of Aβ deposition and related biomarkers. Expanding the application of these staging systems to diverse populations and integrating additional biomarkers, such as cerebrospinal fluid (CSF) and advanced magnetic resonance imaging (MRI) metrics, will be crucial. Further efforts should focus on optimizing visual assessment systems to enhance their reliability and adaptability in clinical and research settings.

Based on the regions of Aβ deposition, we employed three region‐based multi‐phase Aβ deposition systems. (1) Villeneuve stage^7^: negative, regional, and widespread; (2) Grothe stage^8^: from stage 0 to stage 4; (3) Mattsson stage^9^: negative, early, intermediate and late. In the latter two methods, images that fail to meet the criteria at any stage are classified as undetermined (UND).

In Villeneuve stage, the determination was based on the deposition of Aβ across seven regions: medial orbitofrontal, rostral anterior cingulate, posterior cingulate, precuneus, rostral middle frontal, superior frontal, and inferior parietal cortices. Individuals without Aβ deposition in any region were classified as negative, while those exhibiting deposition in one to six regions were classified as regional, the individuals with Aβ deposition in seven regions were categorized as widespread.

In the Grothe stage, according to the deposition sequence of Aβ, the brain was divided into four regions: I: temporobasal and frontomedial areas; II: remaining associative neocortex; III: primary sensory‐motor areas and the medial temporal lobe; IV: the striatum. Individuals without Aβ deposition are classified as stage 0, while those with Aβ deposition found only in region I are classified as stage 1. Stages 2–4 were characterized by additional involvement of regions II, III, and IV. Note that due to the limited number of participants in stage 1, participants in stages 1 and 2 were combined into a single stage 1+2 for the analysis.

In the Mattsson stage, brain was divided into three regions: I: lateral orbitofrontal, insula, precuneus, posterior cingulate, medial orbitofrontal, isthmus cingulate; II: bank of superior temporal sulcus, caudal middle frontal, fusiform, inferior temporal, middle temporal, rostral middle frontal, superior frontal, putamen, pars opercularis, pars triangularis, supramarginal, pars orbitalis, superior parietal, lateral occipital, cuneus, frontal pole, inferior parietal, superior temporal, parahippocampal, rostral anterior cingulate; III: lingual, paracentral, pericalcarine, postcentral, precentral. Individuals without Aβ deposition in the aforementioned regions were classified as negative, those with Aβ deposition localized solely in region I were categorized as early, while individuals exhibiting Aβ deposition in both regions I and II were classified as intermediate, and individuals with Aβ deposition in all three regions were categorized as late.

In the judgment of tau‐PET results, negative indicates that tau deposition was not found in the brain, MTL+ indicates tau deposition predominantly confined to the medial temporal lobe; MOD+ represents moderate tau deposition extending beyond the medial temporal lobe to include additional cortical regions; HIGH+ reflects high levels of tau deposition with widespread involvement of cortical regions.

### 2.7 Functional and structural brain metrics

Participants were scanned to acquire resting‐state functional MRI (fMRI) and T1‐weighted structural MRI data in a 3.0 Tesla scanner (SIEMENS MAGNETOM Prisma 3.0 T, Siemens, Erlangen, Germany) at the Shanghai Sixth People's Hospital affiliated with Shanghai Jiaotong University School of Medicine. Resting‐state fMRI data were used to measure the fractional amplitude of low‐frequency fluctuations (fALFF) and connectivity in default mode network (DMN) using predefined regions of interest.^12^, ^22^ T1‐weighted MRI data was analyzed to assess hippocampus and hippocampal subfield volumes. See details in Supplementary Methods.

### 2.8 Peripheral blood biomarkers

Plasma Aβ42, Aβ40, P‐tau181, total tau, and NfL levels were measured using Simoa technology. APOE genotype was determined using a single nucleotide polymorphism assay. Detailed methods are provided in Supplementary Methods.

### 2.9 Statistical analysis

Statistical analyses were performed using R version 4.4.1. Continuous variables were expressed as medians with interquartile ranges, and categorical variables were presented as proportions. Comparisons of demographic characteristics, neuropsychological performance, Aβ‐PET stage distributions, and biomarker levels across groups (normal cognition [NC], SCD, MCI, and dementia) were conducted using the Kruskal–Wallis test for continuous variables and the chi‐squared test for categorical variables. Biomarkers (plasma and imaging) across different stages in the three staging systems were compared using the Kruskal–Wallis test, with pairwise comparisons for plasma biomarkers conducted using the Dwass‐Steel‐Critchlow‐Fligner method. Correlations between Aβ deposition (SUVR levels) and neuropsychological test scores at different stages of the staging systems were analyzed with adjustments for age, sex, and education. False discovery rate (FDR) correction was applied to account for multiple comparisons. Statistical significance was set at *p* < 0.05.

## 3 RESULTS

### 3.1 Demographic information and neuropsychological test performance

**FIGURE 1:**
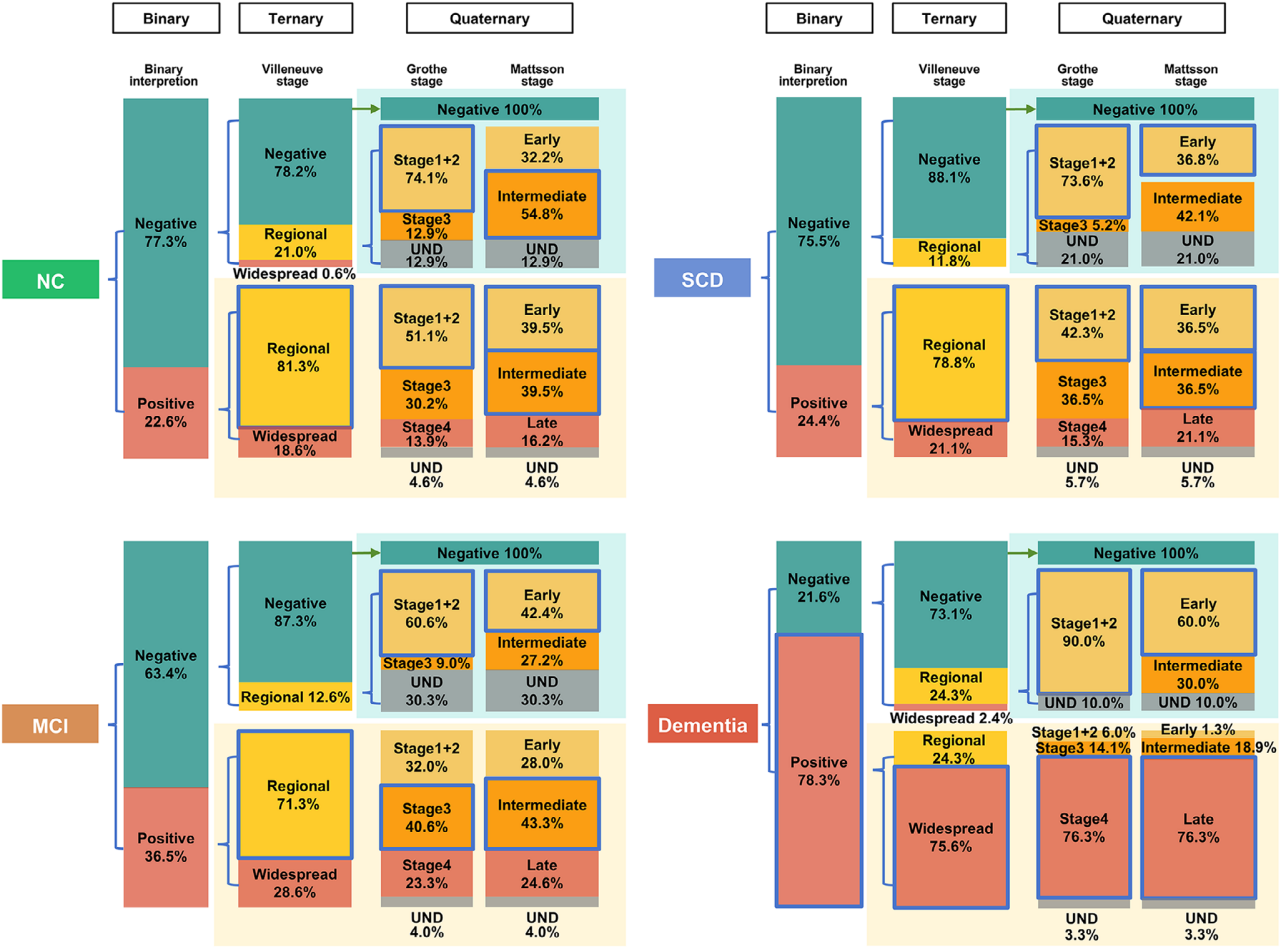
Aβ‐PET visual classification across different cognitive stages. Aβ‐PET imaging is visually interpreted across four cognitive stages: NC, SCD, MCI, and dementia. The classification methods include a binary approach, which categorizes Aβ‐PET images into negative and positive; a ternary classification based on the Villeneuve stage system; and a quaternary classification incorporating Grothe stage and Mattsson stage system. Aβ, amyloid‐beta; MCI, mild cognitive impairment; NC, normal cognition; PET, positron emission tomography; SCD, subjective cognitive decline.

The study included 1002 participants: 190 with NC, 213 with SCD, 410 with MCI, and 189 with dementia. Median ages ranged from 64 (NC) to 68 (dementia) years, with significant group differences (*p* < 0.001). Years of education also declined with disease progression (NC: 13 years; dementia: 9 years, *p* < 0.001). Sex distribution was similar across groups (*p* = 0.632). Aβ‐PET positivity increased from 22.63% (NC) to 78.31% (dementia, *p* < 0.001), see Table 1, Figure 1.

**TABLE 1:** Demographics and distribution of Aβ deposition stages

Parameter	NC (*n* = 190)	SCD (*n* = 213)	MCI (*n* = 410)	Dementia (*n* = 189)	Statistic	*p‐*value
Age, years	64 (58.5,70.75)	65 (57,69)	67 (62,71)	68 (62,74)	*H* = 33.73	< 0.001
Education years	13 (10,15)	12.5 (11,15)	11 (9,14)	9 (6.5,12)	*H* = 97.04	< 0.001
Sex (female [%])	113 (59.47 %)	138 (64.79 %)	249 (60.73 %)	112 (59.26 %)	*χ* ^2^ = 1.72	0.632
Aβ‐PET binary result (positive [%])	43 (22.63%)	52 (24.41%)	150 (36.59%)	148 (78.31%)	*χ* ^2^ = 163.85	< 0.001
**Villeneuve stage**						
Negative (*n* [%])	115 (60.53 %)	142 (66.67 %)	227 (55.37 %)	30 (15.87 %)		
Regional (*n* [%])	66 (34.74 %)	60 (28.17 %)	140 (34.15 %)	46 (24.34 %)		
Widespread (*n* [%])	9 (4.74 %)	11 (5.16 %)	43 (10.49 %)	113 (59.79 %)	*χ* ^2^ = 305.88	<0 .001
**Grothe stage (*n* [%])**						
Stage 0 (*n* [%])	115 (60.53 %)	142 (66.67 %)	227 (55.37 %)	30 (15.87 %)		
Stage 1+2 (*n* [%])	45 (23.68 %)	36 (16.90 %)	68 (16.59 %)	18 (9.52 %)		
Stage 3 (*n* [%])	17 (8.95 %)	20 (9.39 %)	64 (15.61 %)	22 (11.64 %)		
Stage 4 (*n* [%])	7 (3.68 %)	8 (3.76 %)	35 (8.54 %)	113 (59.79 %)		
UND (*n* [%])	6 (3.16 %)	7 (3.29 %)	16 (3.90 %)	6 (3.17 %)	*χ* ^2^ = 353.32	<0 .001
**Mattsson stage**						
Negative (*n* [%])	115 (60.53 %)	142 (66.67 %)	227 (55.37 %)	30 (15.87 %)		
Early (*n* [%])	27 (14.21 %)	26 (12.21 %)	56 (13.66 %)	8 (4.23 %)		
Intermediate (*n* [%])	34 (17.89 %)	27 (12.68 %)	74 (18.05 %)	32 (16.93 %)		
Late (*n* [%])	8 (4.21 %)	11 (5.16 %)	37 (9.02 %)	113 (59.79 %)		
UND (*n* [%])	6 (3.16 %)	7 (3.29 %)	16 (3.90 %)	6 (3.17 %)	*χ* ^2^ = 331.72	<0 .001

Abbreviations: Aβ, amyloid beta; MCI, mild cognitive impairment; NC, normal cognitive; PET, positron emission tomography; SCD, subjective cognitive decline; UND, undetermined.

Significant differences in neuropsychological test performance were observed across all groups (*p* < 0.001). Progressive declines were evident across functional (ADL, FAQ), cognitive (MoCA‐B, ACE III), memory (AVLT, BVMT), language (AFT, BNT), and executive/spatial domains (STT‐A and STT‐B, CaST, SCWT, ST, JLO) from NC to SCD, MCI, and dementia groups. See Table S1.

### 3.2 Distribution of multi‐phase Aβ deposition systems

Significant group differences were observed in Aβ staging across all systems (*p* < 0.001).

In the Villeneuve system, the Negative stage comprised 60.53% of NC, 66.67% of SCD, 55.37% of MCI, and 15.87% of Dementia participants. The Widespread stage was most prevalent in Dementia (59.79%), followed by MCI (10.49%), SCD (5.16%), and NC (4.74%).

In the Grothe system, 60.53% of NC, 66.67% of SCD, 55.37% of MCI, and 15.87% of Dementia participants were in stage 0. Stage 4 dominated in Dementia (59.79%) but was low in NC (3.68%), SCD (3.76%), and MCI (8.54%) participants.

In the Mattsson system, the Negative stage included 60.53% of NC, 66.67% of SCD, 55.37% of MCI, and 15.87% of Dementia participants, while the Late stage accounted for 59.79% of Dementia, 9.02% of MCI, 5.16% of SCD, and 4.21% of NC participants.

Across all systems, NC and SCD were clustered in earlier stages, while Dementia was concentrated in advanced stages, with MCI reflecting a transitional role. See Table 1, Figure 1.

### 3.3 Pathological biomarkers in multi‐phase Aβ deposition systems

**FIGURE 2:**
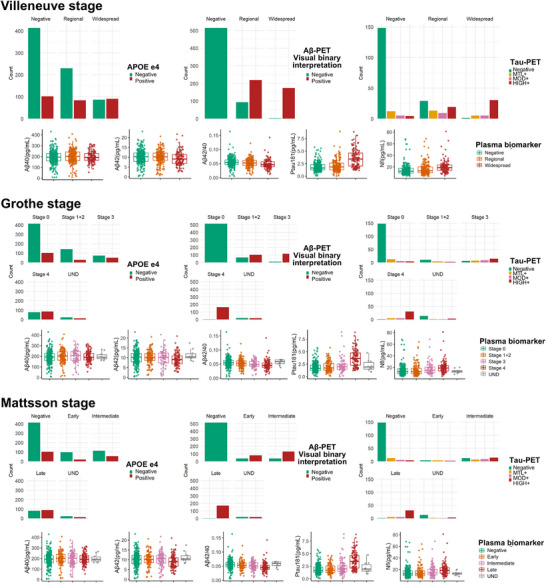
Biomarker levels across different Aβ deposition stages in various staging systems. Biomarker levels are shown across different Aβ deposition stages in various staging systems, including APOE ε4 allele status, Aβ‐PET binary classification results, and tau‐PET visual staging of tau deposition. Biomarkers include plasma Aβ40, Aβ42, phosphorylated tau at threonine 181 (p‐tau181), and NfL. Aβ, amyloid‐beta; APOE, apolipoprotein E; NfL, neurofilament light chain; PET, positron emission tomography.

APOE ε4 carriers increased in advanced stages across all systems, exceeding 50% in Widespread, stage 4, and Late stages (*p* < 0.001). Aβ‐PET positivity approached 100% in advanced stages. Tau‐PET HIGH+ cases also rose significantly in later stages. Plasma Aβ42 levels and Aβ42/Aβ40 ratios declined, while P‐tau181 and NfL levels increased with disease progression (*p* < 0.001). These changes were most pronounced in advanced stages (e.g., Villeneuve's Widespread), with similar trends in Grothe and Mattsson systems. See Figure 2, Table S2, and S3.

### 3.4 The DMN functions in multi‐phase systems

**FIGURE 3:**
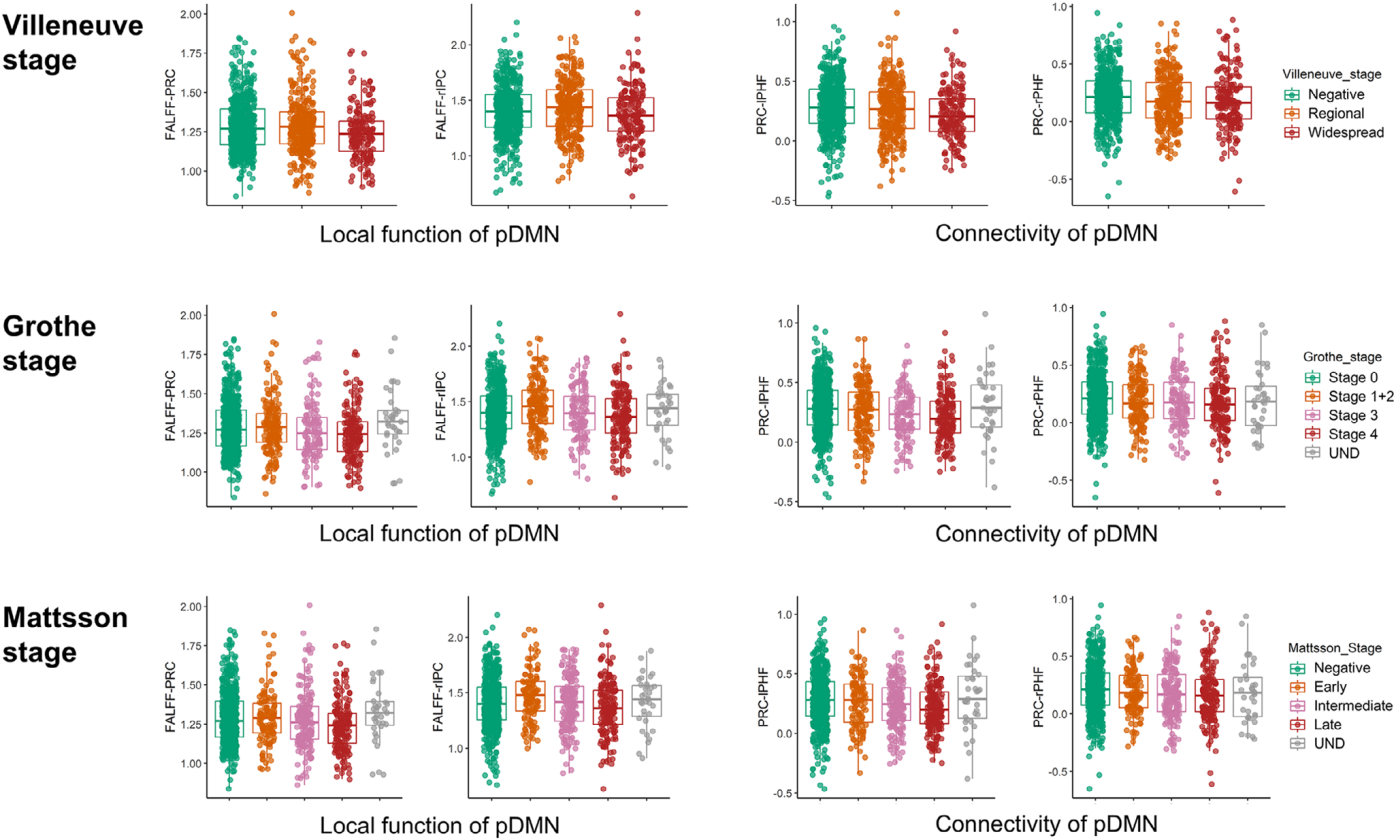
Regional function and functional connectivity of the posterior default mode network across different Aβ deposition stages. Differences in regional function and functional connectivity of the pDMN are observed across various Aβ deposition stages. Post hoc analysis, adjusted for multiple comparisons, reveals significant differences primarily between the earliest and latest stages. Aβ, amyloid‐beta; lPHF, left parahippocampal gyrus; pDMN, posterior default mode network; PRC, precuneus; rPHF, right parahippocampal gyrus.

Local functional activity (fALFF) and posterior DMN connectivity showed stage‐dependent changes. The precuneus (PRC) and right intraparietal cortex (rIPC) fALFF values declined in advanced stages. Connectivity between PRC with left and right parahippocampal formation (lPHF and rPHF) also decreased significantly in later stages. See Figure 3, Tables S4 and S5.

### 3.5 Volume of hippocampal subfields in multi‐phase systems in the overall cohort

**FIGURE 4:**
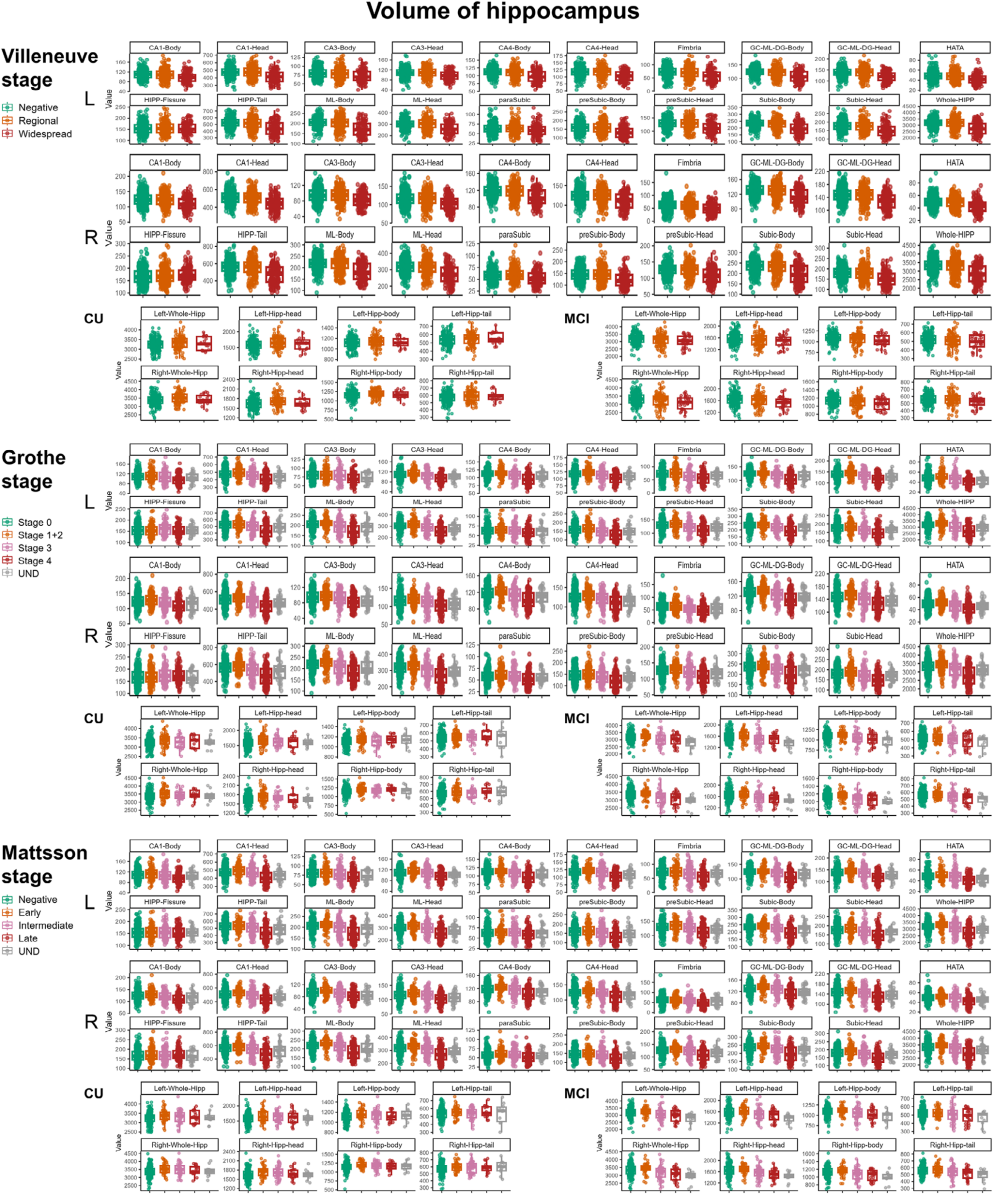
Volume of the hippocampus and hippocampal subregions across different Aβ deposition stages. Differences in hippocampal and hippocampal subregion volumes are observed across various Aβ deposition stages. Overall, hippocampal and subregional volumes tend to decrease as Aβ deposition progresses. In the cognitively unimpaired (including NC and SCD) population, an increase in hippocampal volumes is observed during deposition stages. This trend diminished in MCI where hippocampal volume declined after a slight increase in the early stages. Aβ, amyloid‐beta; CU, cognitively unimpaired; Hipp, hippocampus; MCI, mild cognitive impairment; NC, normal cognition; SCD, subjective cognitive decline.

In the Villeneuve, Grothe, and Mattsson systems, hippocampal subfield volumes (e.g., CA1, CA3, CA4, ML‐HP) significantly decreased with advancing Aβ‐PET stages (*p* < 0.001). In the overall cohort analysis, a slight biphasic trend was observed, with an initial volume increase during early stages—particularly in the two quaternary systems, Grothe stage 1+2 and Mattsson Early stages—followed by a subsequent decrease. The hippocampal fissure remained stable across all stages (*p* > 0.05). See Figure 4, Tables S6–S8.

### 3.6 Hippocampal volume in multi‐phase systems in different cognitive states

In cognitively unimpaired individuals (NC and SCD), hippocampal volume exhibited compensatory increases with advancing Aβ deposition stages. Both left and right hippocampi showed volume increases in deposition‐positive stages across the Villeneuve system and the quaternary Grothe and Mattsson systems.

In individuals with MCI, hippocampal volume increases were only observed in the quaternary systems during early Aβ deposition stages (Grothe stage 1+2 and Mattsson Early stages). These findings suggest a stage‐dependent biphasic pattern, where compensatory changes are more prominent in early stages but diminish as the disease progresses.

See Figure 4, Tables S9, and S10.

### 3.7 Correlation between Aβ deposition and cognitive function in multi‐phase systems

**FIGURE 5:**
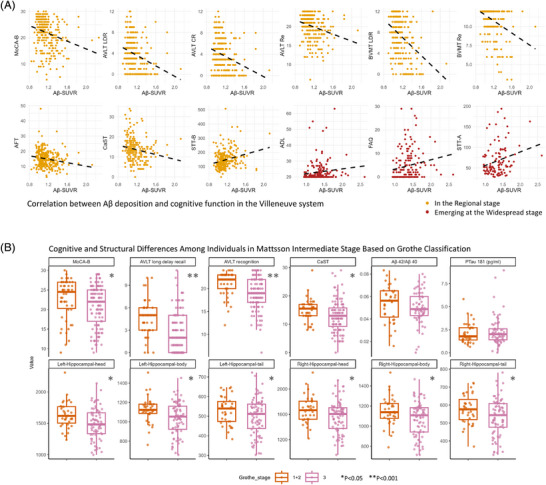
Relationship between Aβ deposition levels and functional and cognitive abilities in the Villeneuve stage‐based classification system. (A) Correlation between Aβ‐SUVR and neuropsychological test performance in Villeneuve stages, adjusted for age, sex, and years of education. The yellow dots represent correlations at the Regional stage, and the red dots represent emerging correlations at the Widespread stage. In the Regional stage, Aβ deposition levels are inversely associated with general cognitive function (MoCA‐B), language function (AFT), executive function (CaST; STT‐B), and memory function (LDR, CR, Re, recognition in AVLT, LDR, and Re in BVMT). In the Widespread stage, Aβ deposition levels are inversely associated with functional activity ability (ADL, FAQ), general cognitive function (MoCA‐B), executive function (CaST, STT‐A, STT‐B), and memory function (LDR and CR in AVLT; LDR and Re in BVMT). (B) Differences between the two quaternary systems. Of the individuals classified as Mattsson Intermediate stage, some were classified as Grothe stages 1+2, and others were classified as stage 3. Comparisons between the two groups showed significant differences in cognitive function and hippocampal volume. However, no significant differences were observed in plasma biomarkers. ADL, activities of daily living; AFT, Animal Fluency Test; AVLT, Auditory Verbal Learning Test; Aβ, amyloid‐beta; BVMT, Brief Visuospatial Memory Test; CaST, Category Switching Test; CR, cued recall; FAQ, Functional Activities Questionnaire; LDR, long delay recall; MoCA‐B, Montreal Cognitive Assessment‐Basic; STT‐A, Shape Trail Test Part A; STT‐B, Shape Trail Test Part B; SUVR, standardized uptake value ratio.

In the Villeneuve system, significant correlations were observed between Aβ deposition and cognitive performance, particularly in the Regional and Widespread stages. Higher Aβ levels were associated with poorer scores on MoCA‐B, AVLT, BVMT, and other cognitive tests (adjusted *p* < 0.05), with functional impairments (ADL, FAQ) becoming evident in the Widespread stage. See Figure 5, Table S11.

In the Grothe system, negative correlations between Aβ deposition and cognition were significant in later stages (stage 3 and stage 4). Stage 4 demonstrated the strongest associations, affecting multiple cognitive domains and functional measures (adjusted *p* < 0.05). Similarly, in the Mattsson system, cognitive decline was linked to Aβ levels in the Intermediate and Late stages, with the Late stage showing the most pronounced deficits in both cognitive and functional tests (adjusted *p* < 0.05). See Figure 5, Tables S12, and S13.

### 3.8 Distribution differences in quaternary systems

Discrepancies were noted between the two quaternary staging systems (Grothe and Mattsson). Among 167 individuals in the Mattsson Intermediate stage, 50 were categorized as Grothe stage 1+2, and 117 as stage 3. See Table S14.

A comparison of cognitive function and hippocampal volumes in Mattsson Early stage participants showed significant differences between those classified as Grothe stages 1+2 and stage 3. Grothe stage 3 participants had lower MoCA‐B scores (*p* = 0.047, effect size = 0.19) and poorer memory and executive function, including AVLT long delay recall (*p* < 0.001, effect size = 0.42), AVLT recognition (*p* < 0.001, effect size = 0.42), and CaST (*p* = 0.006, effect size = 0.29). See Figure 5, Table S15.

Hippocampal volumes were also smaller in Grothe stage 3 participants, particularly in the left hippocampal head (*p* = 0.005, effect size = 0.34), body (*p* = 0.009, effect size = 0.31), and right hippocampal subfields (head, body, tail; *p* < 0.05). Plasma biomarkers showed no significant differences (*p* > 0.05). See Figure 5, Table S15.

## 4 DISCUSSION

In this study, we adapted three multi‐phase Aβ staging systems for visual assessment and used them to examine associations between Aβ deposition, cognition, neurodegeneration, and plasma biomarkers. Our findings revealed a progressive pattern of Aβ expansion and its associated pathological and clinical features.

In the Villeneuve staging system, Aβ deposition was observed to progress from the Regional stage to Widespread stage. A small proportion of NC and SCD participants were found in the Regional stage, suggesting that early Aβ pathology can occur prior to noticeable cognitive decline. Similarly, the Grothe and Mattsson systems demonstrated progressive shifts in stage distribution with increasing disease severity. Most NC and SCD participants were concentrated in the early stages (negative or stage 0), while MCI participants exhibited intermediate distributions, reflecting their transitional role.^23^ The dementia group, however, was heavily represented in the most advanced stages (stage 4 in Grothe and Late in Mattsson), consistent with significant neurodegeneration and cognitive decline.^24^, ^25^ The consistent progression patterns across these three systems reinforce their robustness and potential for tracking the continuum of AD development.

Notably, the presence of participants in the UND category in the Grothe and Mattsson systems suggests that some individuals exhibit Aβ deposition patterns that deviate from the typical progression. This may represent distinct AD subtypes or reflect heterogeneity in Aβ‐related neurodegenerative processes.^26^, ^27^, ^28^ In addition, we observed inconsistencies among the staging systems, particularly between Mattsson's Intermediate stage and Grothe's stages 1+2 or stage 3, likely due to differences in defining early regional Aβ deposition. However, these differences reflect complementary perspectives on Aβ spatial progression rather than contradictions. Supplementary biomarker assays and the combined optimization of imaging region segmentation and diagnostic thresholds may aid in the definitive diagnosis of borderline cases. As these technical frameworks undergo iterative evolution, establishing an integrated assessment framework incorporating multimodal biomarker integration analysis and standardized thresholding protocols could effectively mitigate diagnostic bias caused by criteria heterogeneity in clinical practice and cohort studies.

The prior longitudinal analysis from large cohorts indicates that over a 2‐ to 6‐year period, approximately 10%–20% of participants initially classified as Aβ‐negative can progress to early or more advanced Aβ deposition stages. Meanwhile, more than half of those with early or intermediate Aβ deposition eventually transition to higher stages.^9^ These findings highlight the importance of identifying individuals at early stages of deposition for timely intervention. Future efforts within the C‐PAS cohort will incorporate longitudinal imaging to track transitions among Aβ stages and further validate the clinical relevance of these staging systems.

In advanced Aβ‐PET stages, pathological biomarkers such as APOE ε4 genotype, tau‐PET positivity, and plasma biomarkers demonstrated clear stage‐dependent changes. A higher prevalence of APOE ε4 carriers was observed in advanced stages (e.g., Villeneuve's Widespread, Grothe's stage 4, and Mattsson's Late stages), consistent with its role in accelerating Aβ deposition.^29^, ^30^ Similarly, tau pathology, as indicated by tau‐PET, was significantly more prevalent in later stages, aligning with the amyloid cascade hypothesis.^7^ Plasma biomarkers (Aβ42, Aβ42/Aβ40 ratio, P‐tau181, NfL) exhibited stage‐specific divergence, with Aβ42 levels and Aβ42/Aβ40 ratio declining as pathology progressed, while P‐tau181 and NfL levels showed concurrent elevation.^31^, ^32^

Plasma biomarkers showed limited sensitivity in early and intermediate stages of Aβ deposition. They appear to perform better in later stages, when Aβ pathology is more extensive. These findings underscore the need for further validation in NC and SCD populations before applying plasma markers for early detection. Studies indicate that plasma biomarkers can predict Aβ deposition rates,^33^ making longitudinal validation in preclinical populations particularly valuable. Despite the current limitations in establishing a consistent correspondence between plasma biomarkers and specific AD pathology stages, these biomarkers remain a promising detection strategy.^25^ Future efforts may involve refining assays for novel p‐tau isoforms and integrating plasma‐based markers with imaging to enhance early detection.

The DMN is highly sensitive to early Aβ deposition,^34^, ^35^ particularly in key regions.^12^ Analyses revealed that specific DMN nodes, such as the PRC and rIPC, show significant functional changes during Aβ accumulation. Across all three staging systems, PRC fALFF values significantly declined at advanced stages, reflecting its vulnerability as a DMN hub.^36^, ^37^ In the Grothe and Mattsson systems, detailed staging revealed transient PRC functional increases in early stages, possibly reflecting compensatory mechanisms to maintain network function.^38^ However, as Aβ deposition progresses, this compensation diminishes. This finding highlights the sensitivity of multi‐phase methods in detecting early functional changes. At the network level, posterior DMN connectivity declined significantly in advanced stages, while anterior connectivity remained relatively preserved. This reflects a functional dissociation within the DMN, with the posterior region being more susceptible to Aβ deposition.^39^, ^40^

Analysis of hippocampal subfield volumes across the three staging systems reveals how Aβ pathology progression influences neurodegeneration. Consistently across the three systems, we observed significant volume reductions in advanced stages of Aβ deposition, particularly in subfields such as CA1, CA3, CA4, and ML‐HP, which are highly susceptible to Aβ‐related damage.^41^, ^42^, ^43^ This progressive atrophy aligns with the expected trajectory of hippocampal degeneration in AD and underscores the utility of these staging systems in capturing structural changes associated with disease progression. The stability of the hippocampal fissure across all stages suggests that certain hippocampal regions may be less responsive to Aβ‐related pathology, potentially due to their distinct anatomical and functional roles.^43^, ^44^

In cognitively unimpaired individuals, the biphasic pattern was observed, characterized by an increase in hippocampal volumes as Aβ began to deposit in the brain.^45^ This biphasic trajectory may reflect early compensatory processes or inflammatory responses before the onset of significant neurodegeneration.^46^, ^47^ In the MCI population, hippocampal volume changes show differences in sensitivity among the staging systems. The Grothe and Mattsson systems captured an initial volume increase in early stages, followed by a marked decline in later stages. The Villeneuve system also captured this pattern to some extent but demonstrated lower sensitivity in detecting early changes compared to the Grothe and Mattsson systems. Findings from hippocampal and subfield volumes support the utility of multi‐phase Aβ staging systems in providing sensitive detection of subtle neurodegenerative changes.

The relationship between Aβ deposition and cognition across staging systems reflects its contribution to cognitive decline at different pathological stages. Notably, the Villeneuve staging system demonstrated unique sensitivity to the localized effects of Aβ deposition in the Regional stage. At this stage, significant negative correlations were observed between Aβ deposition and cognitive abilities, particularly in memory, executive, and language functions. Furthermore, in the Widespread stage, the system continued to show strong negative correlations between Aβ pathology and cognitive performance, as well as impairments in daily activities and functional abilities. The quaternary Grothe and Mattsson systems demonstrated associations between Aβ deposition and cognitive function in the middle‐to‐late stages, suggesting that early mild Aβ deposition is insufficient to directly cause cognitive decline.

Overall, we adapted and validated three multi‐phase Aβ deposition staging systems, applying them to establish visual grading methods. From the results, mid‐to‐late stages across all three staging systems were closely associated with significant cognitive decline, hippocampal atrophy, and altered DMN function. However, in the early stages, some individuals already exhibit cognitive impairment or tau deposition positivity. In the preclinical stages, certain hippocampal subfields exhibited nonlinear changes, with initial volume increases followed by subsequent declines. This suggests early compensatory mechanisms triggered by Aβ deposition and underscores the need to shift the intervention window earlier to the onset of Aβ accumulation. The plasma biomarkers examined in this study showed stage‐dependent changes within the staging systems. Although their sensitivity to early and intermediate Aβ deposition was limited, they may still be useful for detecting widespread Aβ pathology. Among the three staging systems used in this study, the ternary Villeneuve system proved suitable for populations in the mid‐stages of the disease, while the quaternary Grothe and Mattsson systems were better suited for early‐stage individuals. Notably, if different centers adopt divergent staging frameworks, patients with borderline or focal Aβ deposition might face potential inconsistencies in therapeutic strategies. Future development of consensus guidelines or mapping between staging systems may help unify staging decisions across diverse settings.

In conclusion, this study underscores the clinical and pathological significance of multi‐phase Aβ staging systems, demonstrating their utility in identifying disease stages, tracking pathological progression, and elucidating the mechanisms underlying cognitive decline and neurodegeneration in AD. Future research should focus on further optimizing these staging systems to address the heterogeneity of Aβ deposition patterns and expand their applicability to personalized diagnostic and therapeutic strategies.

### 4.1 Limitations

Several limitations should be acknowledged. The cross‐sectional design limits the ability to evaluate temporal changes in Aβ deposition stages and their relationship to disease progression. Longitudinal studies are needed to validate the dynamic progression patterns observed in this study. The cohort consists exclusively of Chinese participants, which may restrict the generalizability of the findings to other populations. Regional and genetic differences in Aβ pathology and AD progression warrant further validation in diverse cohorts. Future research should focus on longitudinal designs, multi‐modal biomarker integration, and validation across diverse populations to refine the utility of multi‐phase Aβ staging systems in clinical and research settings.

## CONFLICT OF INTEREST STATEMENT

The authors declare that they have no conflict of interest. Author disclosures are available in the Supporting Information.

## CONSENT STATEMENT

The study was reviewed and approved by the Ethics Committee of Shanghai Sixth People's Hospital (approval number 2019‐041). It was performed in accordance with the principles of the Declaration of Helsinki. All participants provided written informed consent to participate in the study.

## Supporting information



Supporting Information

Supporting Information

Supporting Information
