# Supplementary material for: Beyond binary classification: Comparing three region‐based multi‐phase Aβ staging systems
Source: Alzheimers Dement. 2025 May 19;21(5):e70253. doi: 10.1002/alz.70253 (PMC12089080; doi:10.1002/alz.70253)
Supplement: Supplementary file 1 — Supporting Information [file ALZ-21-e70253-s002.docx]

**Beyond Binary Classification: Comparing Three Region-Based Multi-Phase Aβ Staging Systems**

## Supplementary Methods

**Diagnostic criteria for cognitive impairment**

The recruitment process for Subjective Cognitive Decline (SCD) participants adhered to the guidelines established by the SCD-initiative (SCD-I) framework^1^. Individuals were categorized as SCD if they fulfilled the following criteria: they self-reported a subjective cognitive decline (perceived as being worse compared to their peers) accompanied by related concerns, experienced the onset of this decline within the 5 years preceding the interview, and did not demonstrate notable objective cognitive impairment, as their neuropsychological test results did not fulfill the criteria for Mild Cognitive Impairment (MCI).

The diagnostic criteria for MCI adhere to the Petersen criteria^2^, which include: 1) self-reported cognitive decline observed by the individual, family, or an informant; 2) cognitive assessment scores that are at least 1.5 standard deviations below the mean for the individual’s age and educational background (MoCA-B was used in this study to assess); 3) the cognitive function remains predominantly intact; 4) no significant impairment in daily activities; 5) exclusion of dementia or any other physical or mental health conditions that might affect cognitive function.

The diagnosis of dementia conforms to the 2011 National Institute on Aging-Alzheimer’s Association (NIA-AA) criteria for probable AD dementia^3^.

**Aβ-positron emission tomography (PET) image acquisition and processing**

Cerebral Aβ deposition was visualized with 18F-florbetapir PET. The PET imaging was performed using the Siemens Biograph mCT FlowMotion PET/CT system located at the PET Center of Huashan Hospital, affiliated with Fudan University. The imaging protocol involved administering 10 mCi (370 MBq) of the tracer intravenously, followed by a 50-minute rest period before conducting a 20-minute imaging session. After image acquisition, the PET images were reconstructed using the ordered subset expectation maximization algorithm (OSEM) with weighted attenuation, which included attenuation correction. Following this, the reconstructed PET images were fused with CT images.

Calculation of Standardized Uptake Value Ratio (SUVR): SPM12 (http://fil.ion.ucl.ac.uk/spm/) in MATLAB 2016b (MathWorks Inc) was used to preprocess the PET data. PET images of each participant were aligned with their T1-MRI images. The unified segmentation of SPM was used to segment T1-MRI images into gray matter, white matter and cerebrospinal fluid probability maps. PET images were warped into Montreal Neurological Institute space using the transformed parameter space obtained by MRI segmentation. To improve the signal-to-noise ratio, the normalized PET images were smoothed using an 8-mm isotropic Gaussian kernel. The SUVR of the cerebral cortex was obtained using the cerebellum as the reference region.

**Tau-PET image acquisition**

The Tau-PET/CT imaging was conducted at the PET Center of Huashan Hospital, Fudan University, utilizing the Siemens Biograph 64 PET/CT scanner with an 18F-MK6240 tracer. The participants underwent an intravenous injection of 18F-MK6240 at a dosage of 5.55 MBq/kg body weight. They rested in a quiet environment for 90 minutes before the 20-minute brain PET/CT imaging session. Brain PET/CT imaging consists of a low-dose CT scan for the purpose of attenuation correction. A filtered back-projection algorithm was used for image reconstruction.

**Resting State Functional Magnetic Resonance Imaging (Rs-fMRI) image acquisition and processing**

Participants were asked to keep their eyes closed, remain relaxed without falling asleep, and minimize movements during the scans. To minimize head motion during scanning, the participants’ heads were immobilized using a foam cushion with a pillow placed under the head. Transverse plane echo planar imaging (EPI) sequences were used for image acquisition. The specific parameters were as follows: repetition time=800 ms, echo time=37 ms, flip angle=52°, matrix size=104×104, field of view=208 mm×208 mm, slice number=72 slices, slice thickness=2 mm, and voxel size=2 mm×2 mm×2 mm. The imaging procedure produced 488 slices. SPM12 (http://fil.ion.ucl.ac.uk/spm/) and RESTplus (http://restfmri.net/forum/restplus) were used to preprocess the data.

The pre-processing of the image includes the following steps: discarded the first 30 time points, realign the head motion (participants with head movement exceeding 3 mm or 3° were excluded), spatially normalized to the Montreal Neurological Institute (MNI) space and resampled to 3 mm isotropic voxels, linear and quadratic trends of the time-series signals were removed, the sign (including white matter, cerebrospinal fluid, global mean signal, and Friston-24 motion parameters) were. regressed out. The pre-processed image was acquired by the above steps.

After smoothing the pre-processed images by Full Wave at Half Maximum (FWHM) 6 mm, the fractional amplitude of low-frequency fluctuations (fALFF) in the low-frequency range (0.01-0.08 Hz) was calculated. The fALFF values for each voxel were normalized by dividing them by the overall average fALFF value for all voxels in the brain to generate mfALFF maps for each participant. The fALFF values for 10 region of interest (ROI)s of default mode network (DMN) were extracted. The 10 ROIs of DMN were used for ROI-to-ROI connectivity analysis. The reference time series for each ROI was calculated by averaging the time series for each ROI and then calculating the time series correlation coefficient between each pair of ROIs. Correlation coefficients were normalized using the Fisher Z-transform.

We utilized a ROI-based approach to define the DMN. Based on the research conducted by Ereira S et al^4^,and verified in our previous studies^5^, the DMN was defined using the following 10 ROIs: the anterior medial prefrontal cortex (amPFC), the precuneus(PRC), the left and right intraparietal cortex (lIPC and rIPC), the ventromedial prefrontal cortex (vmPFC), the dorsomedial prefrontal cortex (dmPFC), left and right lateral temporal cortex (lLTC and rLTC), and left and right parahippocampal formation (lPHF and rPHF). Each ROI was identified by delineating a sphere with an 8-mm radius centered on the designated coordinate. Rs-fMRI image acquisition and processing see Supplementary Methods in Supplementary Materials.

**Hippocampus and subfield region volume**

Whole-brain high-resolution T1-weighted (T1w) images were acquired with a 3D Magnetization Prepared Rapid Gradient Echo (MPRAGE) sequence (TR=2.56 ms; TE=3000 ms, flip angle=7, matrix size=320x320, 208 sagittal slices, voxel size = 0.8 × 0.8 × 0.8 mm2). High-resolution T1w images were first visually inspected to exclude artifacts induced by severe head motions. The images were then preprocessed using FreeSurfer 7.3.2. (http://surfer.nmr.mgh.harvard.edu) via the recon-all pipeline. Preprocessing steps included image registration, skull stripping, segmentation of brain tissues, tessellation of gray-white matter boundaries, topology correction, and surface deformation. After brain segmentation and surface reconstruction, automated hippocampal subfields segmentation was performed using the module implemented in FreeSurfer^6^. This process consistently generated hippocampal subfield volumes across all participants and yielded 12 subfield regions for each hemisphere: CA1, CA2/3, CA4, molecular layer, granule cell layer of the dentate gyrus (GC-ML-DG; FreeSurfer naming convention), hippocampal tail, subiculum, presubiculum, parasubiculum, fimbria, hippocampal fissure, and the hippocampal amygdala transition area (HATA). To avoid mislabeling of subfields, particularly in participants with severe hippocampus atrophy, the segmented volumes were visually inspected for outliers following Enhancing Neuro Imaging Genetics through Meta Analysis (ENIGMA) protocols. This was conducted using visualization QC tool provided by ENIGMA Consortium^7^. After rigorous quality control, the data of 728 participants were used for subsequent analysis.

**Peripheral blood biomarkers**

Plasma Aβ42, Aβ40, total-tau (T-tau), phosphorylated-tau181 (P-tau181) and neurofilament light chain (Nfl) as peripheral blood AD biomarkers in 596 subjects. Quanterix's newly developed Simoa technology (single molecule arrays) is utilized for plasma biomarker detection. The two-step reaction method was used for the detection of all five biomarkers. Aβ42 and Aβ40 were measured using the Neurology 3-5028 Plex Assay kit. In the initial phase, 25 μl of beads, 20 μl of detector and 38 μl of sample were diluted with 114 μl of diluent prior to 47 cadences (45 seconds each). Subsequently, 100 μl β-galactosidasestreptavidin (SBG) was added to the reaction mixture and the incubation time was 7 cadences. Finally, measurements were performed by adding 50 μl of reserpine β-D-galactopyranoside (RGP). P-tau 181 Assay Kit V2 (Lot 502923) was used to quantify P-tau 181. In the initial phase, 25 μl of beads, 20 μl of detector and 25 μl of sample were diluted with 75 μl of diluent prior to 47 cadences. The subsequent steps are the same as for Aβ measurements. NF-light Assay Kit (Lot 202700) was used to detect Nfl. In the initial phase, 25 μl of beads, 20 μl of detector and 38 μl of sample were diluted with 114 μl of diluent prior to an incubation period of 47 cadences. Subsequently, 100 μl of SBG was introduced to the reaction mixture, and the incubation time was set at 7 cadences. Finally, Finally, measurements were performed by adding 50 μl of reserpine β-D-galactopyranoside (RGP).^8^. The APOE allele was examined using a single nucleotide polymorphism detection reaction technique assay.

## References

1. Jessen, F. *et al.* Design and first baseline data of the DZNE multicenter observational study on predementia Alzheimer’s disease (DELCODE). *Alzheimers Res Ther* **10**, 15 (2018).

2. Petersen, R. C. Mild cognitive impairment as a diagnostic entity. *J Intern Med* **256**, 183–194 (2004).

3. McKhann, G. M. *et al.* The diagnosis of dementia due to Alzheimer’s disease: recommendations from the National Institute on Aging-Alzheimer’s Association workgroups on diagnostic guidelines for Alzheimer’s disease. *Alzheimers Dement* **7**, 263–269 (2011).

4. Early detection of dementia with default-mode network effective connectivity | Nature Mental Health. https://www.nature.com/articles/s44220-024-00259-5.

5. Cui, L. *et al.* Association of precuneus Aβ burden with default mode network function. *Alzheimers Dement* (2024) doi:10.1002/alz.14380.

6. Iglesias, J. E. *et al.* A computational atlas of the hippocampal formation using ex vivo, ultra-high resolution MRI: Application to adaptive segmentation of in vivo MRI. *Neuroimage* **115**, 117–137 (2015).

7. Sämann, P. G. *et al.* FreeSurfer-based segmentation of hippocampal subfields: A review of methods and applications, with a novel quality control procedure for ENIGMA studies and other collaborative efforts. *Hum Brain Mapp* **43**, 207–233 (2022).

8. Pan, F. *et al.* Integrated algorithm combining plasma biomarkers and cognitive assessments accurately predicts brain β-amyloid pathology. *Commun Med (Lond)* **3**, 65 (2023).
