# Supplementary material for: Beyond binary classification: Comparing three region‐based multi‐phase Aβ staging systems
Source: Alzheimers Dement. 2025 May 19;21(5):e70253. doi: 10.1002/alz.70253 (PMC12089080; doi:10.1002/alz.70253)
Supplement: Supplementary file 2 — Supporting Information [file ALZ-21-e70253-s001.docx]

**Beyond Binary Classification: Comparing Three Region-Based Multi-Phase Aβ Staging Systems**

## Supplementary Tables

**Supplementary Table 1 Neuropsychological test performance**

|  | **NC(n=190)** | **SCD(n=213)** | **MCI(n=410)** | **Dementia(n=410)** | **Statistic** | **P** |
| --- | --- | --- | --- | --- | --- | --- |
| MoCA-B | 27 (25,28) | 26 (24,28) | 23 (20,25) | 13 (9,16) | 561.67 | < .001 |
| ACE-III | 85 (79,89) | 82 (76,87) | 73 (66,80) | 50.5 (41,59) | 540.12 | < .001 |
| ADL | 20 (20,20) | 20 (20,20) | 20 (20,20) | 22 (20,25) | 292.27 | < .001 |
| FAQ | 0 (0,0) | 0 (0,0) | 0 (0,1) | 5 (1,10.25) | 287.76 | < .001 |
| AVLT short delay recall | 6 (5,7) | 6 (4,7) | 2 (1,3) | 0 (0,1) | 481.24 | < .001 |
| AVLT long delay recall | 6 (4,8) | 5 (4,7) | 2 (1,4) | 0 (0,1) | 397.56 | < .001 |
| AVLT recognition | 22 (21,23) | 22 (21,23) | 19 (17,21) | 16.5 (13,18.75) | 343.51 | < .001 |
| BVMT long delay recall | 10 (8,12) | 10 (8,12) | 7 (4,10) | 0 (0,3) | 229.14 | < .001 |
| BVMT recognition | 12 (12,12) | 12 (12,12) | 12 (10,12) | 8 (6,10) | 217.21 | < .001 |
| AFT | 18 (15,21) | 17 (15,20) | 14 (11,16) | 10 (7,12) | 376.97 | < .001 |
| BNT | 25 (23,27) | 25 (23,27) | 22 (19,25) | 20 (16,22) | 160.47 | < .001 |
| STT-A (s) | 43 (37,53) | 45 (36,53) | 52 (42,65) | 75 (57,94.5) | 140.51 | < .001 |
| STT-B (s) | 117 (96,146) | 117 (96.5,142.5) | 148 (120,181.5) | 208 (168.5,247.5) | 178.54 | < .001 |
| CaST | 16 (14,18) | 14.5 (13,18) | 13 (10,15) | 8 (6,11.75) | 150.78 | < .001 |
| SCWT | 24 (23,24) | 24 (23,24) | 23 (22,24) | 21.5 (19,23) | 75.49 | < .001 |
| SCWT-time (s) | 31 (26,39) | 32 (28,39) | 38 (30,48) | 52 (41.5,67) | 102.09 | < .001 |
| ST | 10 (8,12) | 10 (8,12) | 9 (7,11) | 7 (5,9) | 87.75 | < .001 |
| JLO | 22 (19,25) | 21 (19,24) | 20 (17,23) | 18 (13,21) | 53.54 | < .001 |

Note: NC, normal cognitive; SCD, subjective cognitive decline; MCI, mild cognitive impairment; MoCA-B, Montreal Cognitive Assessment-basic; ACE-III, Addenbrooke's Cognitive Examination III; ADL, Activity of Daily Living; FAQ, Functional Activities Questionnaire; AVLT, Auditory Verbal Learning Test; BVMT, Brief Visuospatial Memory Test; AFT, Animal-Animal-Verbal Fluency Test; BNT, Boston Naming Test; STT, Shape Trail Test; CaST, Category Switching Test; SCWT, Stroop Word-Color Interference Task; ST, Silhouettes Test; JOL, Judgement of Line Orientation.

**Supplementary Table 2 APOE and imaging results of different Aβ-PET stages**

|  |  | **APOE** | | **Aβ-PET binary result** | |
| --- | --- | --- | --- | --- | --- |
|  |  | non-e4, (%) | e4 carrier, (%) | negative, (%) | positive, (%) |
| **Villeneuve stage** | Negative | 411(80.27 %) | 101(19.73 %) | 514(100.00 %) | 0(0.00 %) |
|  | Regional | 222(73.75 %) | 79(26.25 %) | 93(29.81 %) | 219(70.19 %) |
|  | Widespread | 86(50.00 %) | 86(50.00 %) | 2(1.14 %) | 174(98.86 %) |
|  |  | χ²=59.99 | P< .001 | χ²=719.86 | P< .001 |
| **Grothe stage** | Stage 0 | 411(80.27 %) | 101(19.73 %) | 514(100.00 %) | 0(0.00 %) |
|  | Stage 1+2 | 138(84.15 %) | 26(15.85 %) | 66(39.52 %) | 101(60.48 %) |
|  | Stage 3 | 72(60.50 %) | 47(39.50 %) | 9(7.32 %) | 114(92.68 %) |
|  | Stage 4 | 78(49.06 %) | 81(50.94 %) | 1(0.61 %) | 162(99.39 %) |
|  | UND | 20(64.52 %) | 11(35.48 %) | 19(54.29 %) | 16(45.71 %) |
|  |  | χ²=80.88 | P< .001 | χ²=758.96 | P< .001 |
| **Mattsson stage** | Negative | 411(80.27 %) | 101(19.73 %) | 514(100.00 %) | 0(0.00 %) |
|  | Early | 96(83.48 %) | 19(16.52 %) | 37(31.62 %) | 80(68.38 %) |
|  | Intermediate | 111(68.52 %) | 51(31.48 %) | 38(22.75 %) | 129(77.25 %) |
|  | Late | 81(49.09 %) | 84(50.91 %) | 1(0.59 %) | 168(99.41 %) |
|  | UND | 20(64.52 %) | 11(35.48 %) | 19(54.29 %) | 16(45.71 %) |
|  |  | χ²=70.78 | P< .001 | χ²=732.13 | P< .001 |
|  |  | **Tau-PET** | | | |
|  |  | negative, (%) | MTL+, (%) | MOD+, (%) | HIGH+, (%) |
| **Villeneuve stage** | Negative | 148(87.57 %) | 12(7.10 %) | 5(2.96 %) | 4(2.37 %) |
|  | Regional | 29(41.43 %) | 13(18.57 %) | 9(12.86 %) | 19(27.14 %) |
|  | Widespread | 1(2.44 %) | 5(12.20 %) | 5(12.20 %) | 30(73.17 %) |
|  |  |  |  | χ²=150.93 | P< .001 |
| **Grothe stage** | Stage 0 | 148(87.57 %) | 12(7.10 %) | 5(2.96 %) | 4(2.37 %) |
|  | Stage 1+2 | 10(62.50 %) | 4(25.00 %) | 0(0.00 %) | 2(12.50 %) |
|  | Stage 3 | 6(17.14 %) | 7(20.00 %) | 8(22.86 %) | 14(40.00 %) |
|  | Stage 4 | 1(2.44 %) | 5(12.20 %) | 5(12.20 %) | 30(73.17 %) |
|  | UND | 13(68.42 %) | 2(10.53 %) | 1(5.26 %) | 3(15.79 %) |
|  |  |  |  | χ²=176.13 | P< .001 |
| **Mattsson stage** | Negative | 148(87.57 %) | 12(7.10 %) | 5(2.96 %) | 4(2.37 %) |
|  | Early | 4(40.00 %) | 4(40.00 %) | 0(0.00 %) | 2(20.00 %) |
|  | Intermediate | 12(29.27 %) | 7(17.07 %) | 8(19.51 %) | 14(34.15 %) |
|  | Late | 1(2.44 %) | 5(12.20 %) | 5(12.20 %) | 30(73.17 %) |
|  | UND | 13(68.42 %) | 2(10.53 %) | 1(5.26 %) | 3(15.79 %) |
|  |  |  |  | χ²=168.93 | P< .001 |

Note: UND indicates "undetermined". In tau-PET results, MTL+ indicates tau deposition predominantly confined to the medial temporal lobe; MOD+ represents moderate tau deposition extending beyond the medial temporal lobe to include additional cortical regions; HIGH+ reflects high levels of tau deposition with widespread involvement of cortical regions.

**Supplementary Table 3 Plasma biomarker levels in different Aβ-PET stages**

|  | **Aβ 40(pg/ml)** | **Aβ 42(pg/ml)** | **Ptau 181(pg/ml)** | **Aβ 42/Aβ 40** | **Nfl(pg/ml)** |
| --- | --- | --- | --- | --- | --- |
| **Villeneuve stage** |  |  |  |  |  |
| Negative | 194.12 (159.41,227.86) | 10.29 (7.91,12.20) | 1.64 (1.28,2.32) | 0.05 (0.05,0.06) | 12.62 (9.53,17.75) |
| Regional | 202.65 (163.1,242.5) | 10.22 (8.47,12.13) | 1.85 (1.28,2.62) | 0.05 (0.04,0.06) | 13.41 (10.01,19.98) |
| Widespread | 191.54 (165.43,228.12) | 9.10 (6.87,11.37) ***ab*** | 3.43 (2.05,4.62) ***ab*** | 0.05 (0.04,0.06) ***ab*** | 18.8 (14.02,23.4) ***ab*** |
| Statistic | 3.34 | 10.03 | 100.86 | 30.06 | 40.01 |
| P | 0.188 | 0.007 | < .001 | < .001 | < .001 |
| **Grothe stage** |  |  |  |  |  |
| Stage 0 | 194.12 (159.41,227.86) | 10.29 (7.91,12.2) | 1.64 (1.28,2.32) | 0.05 (0.05,0.06) | 12.62 (9.53,17.75) |
| Stage 1+2 | 203.1 (162.8,242.1) | 10.17 (8.57,12.15) | 1.74 (1.26,2.67) | 0.05 (0.04,0.06) | 13.01 (9.53,18.21) |
| Stage 3 | 207.61 (163.29,246.22) | 10.18 (7.4,12.36) | 2.01 (1.45,2.52) | 0.05 (0.04,0.06) ***c*** | 15.25 (11.02,20.78) ***c*** |
| Stage 4 | 191.06 (165.18,227.55) | 9.07 (6.87,10.93) ***cd*** | 3.64 (2.3,4.74) ***cde*** | 0.05 (0.04,0.05) ***cd*** | 18.88 (13.93,23.31) ***cd*** |
| UND | 192.11 (177.11,214.62) | 10.44 (9.91,12.02) | 1.94 (1.52,2.83) | 0.06 (0.05,0.07) | 13.69 (11.85,14.34) |
| Statistic | 3.69 | 11.81 | 112.08 | 35.77 | 42.61 |
| P | 0.297 | 0.008 | < .001 | < .001 | < .001 |
| **Mattsson stage** |  |  |  |  |  |
| Negative | 194.12 (159.41,227.86) | 10.29 (7.91,12.2) | 1.64 (1.28,2.32) | 0.05 (0.05,0.06) | 12.62 (9.53,17.75) |
| Early | 203.1 (167.75,240.68) | 10.12 (8.57,11.96) | 1.71 (1.25,2.57) | 0.05 (0.04,0.06) | 12.76 (9.34,18.07) |
| Intermediate | 203.62 (155.77,246.19) | 10.56 (8.29,12.29) | 1.96 (1.39,2.69) | 0.05 (0.04,0.06) | 14.27 (10.95,20.68) |
| Late | 192.02 (164.7,228.46) | 8.97 (6.73,11) ***fgh*** | 3.46 (2.14,4.65) ***fgh*** | 0.04 (0.04,0.05) ***fgh*** | 18.71 (13.57,23.24) ***fgh*** |
| UND | 192.11 (177.11,214.62) | 10.44 (9.91,12.02) | 1.94 (1.52,2.83) | 0.06 (0.05,0.07) | 13.69 (11.85,14.34) |
| Statistic | 3.43 | 13.19 | 108.52 | 37.49 | 39.05 |
| P | 0.33 | 0.004 | < .001 | < .001 | < .001 |

Note: UND indicates "undetermined," and this group is not included in the statistical analysis. The meaning of italicized letters is as follows: In the Villeneuve grading system, a denotes a significant difference compared to the Negative group, and b denotes a significant difference compared to the Regional group. In the Grothe grading system, c denotes a significant difference compared to the Stage 0 group, d denotes a significant difference compared to the Stage 1+2 group, and e denotes a significant difference compared to the Stage 3 group. In the Mattsson grading system, f denotes a significant difference compared to the Negative group, g denotes a significant difference compared to the Early group, and h denotes a significant difference compared to the Intermediate group.

**Supplementary Table 4 Local functions of default mode network nodes in different Aβ-PET stages**

|  | **Stage** | | | | | **Statistic** | **P** |
| --- | --- | --- | --- | --- | --- | --- | --- |
| **Villeneuve stage** | **Negative** | **Regional** | **Widespread** |  |  |  |  |
| FALFF-amPFC | 1.22 (1.1,1.32) | 1.21 (1.12,1.33) | 1.25 (1.13,1.38) |  |  | 4.28 | 0.118 |
| FALFF-PRC | 1.27 (1.17,1.4) | 1.28 (1.17,1.38) | 1.24 (1.13,1.32) |  |  | 12.68 | 0.002 |
| FALFF-lIPC | 1.24 (1.13,1.36) | 1.25 (1.14,1.36) | 1.22 (1.12,1.34) |  |  | 3.97 | 0.137 |
| FALFF-rIPC | 1.4 (1.26,1.55) | 1.44 (1.27,1.6) | 1.36 (1.22,1.52) |  |  | 8.63 | 0.013 |
| FALFF-vmPFC | 0.9 (0.84,0.97) | 0.9 (0.85,0.97) | 0.9 (0.85,0.98) |  |  | 0.16 | 0.921 |
| FALFF-dmPFC | 1.16 (1.06,1.29) | 1.16 (1.07,1.28) | 1.2 (1.08,1.3) |  |  | 2.88 | 0.237 |
| FALFF-lLTC | 1.11 (1.03,1.22) | 1.12 (1.02,1.22) | 1.12 (1.01,1.21) |  |  | 0.06 | 0.97 |
| FALFF-rLTC | 1.13 (1.04,1.24) | 1.13 (1.03,1.24) | 1.11 (1.05,1.23) |  |  | 0.29 | 0.863 |
| FALFF-lPHF | 0.84 (0.8,0.89) | 0.84 (0.8,0.88) | 0.84 (0.8,0.89) |  |  | 1.1 | 0.578 |
| FALFF-rPHF | 0.84 (0.8,0.88) | 0.84 (0.8,0.89) | 0.84 (0.8,0.88) |  |  | 2.24 | 0.327 |
| **Grothe stage** | **Stage 0** | **Stage 1+2** | **Stage 3** | **Stage 4** | **UND** |  |  |
| FALFF-amPFC | 1.22 (1.1,1.32) | 1.21 (1.13,1.33) | 1.22 (1.12,1.33) | 1.24 (1.13,1.39) | 1.21 (1.1,1.36) | 3.77 | 0.288 |
| FALFF-PRC | 1.27 (1.17,1.4) | 1.29 (1.19,1.37) | 1.25 (1.14,1.35) | 1.24 (1.13,1.32) | 1.32 (1.24,1.39) | 11.76 | 0.008 |
| FALFF-lIPC | 1.24 (1.13,1.36) | 1.24 (1.13,1.34) | 1.23 (1.14,1.34) | 1.22 (1.12,1.34) | 1.27 (1.19,1.44) | 1.4 | 0.706 |
| FALFF-rIPC | 1.4 (1.26,1.55) | 1.46 (1.3,1.6) | 1.39 (1.24,1.55) | 1.36 (1.22,1.53) | 1.44 (1.29,1.56) | 11.7 | 0.008 |
| FALFF-vmPFC | 0.9 (0.84,0.97) | 0.92 (0.86,0.98) | 0.9 (0.86,0.97) | 0.9 (0.85,0.97) | 0.86 (0.81,0.93) | 2.21 | 0.53 |
| FALFF-dmPFC | 1.16 (1.06,1.29) | 1.18 (1.1,1.28) | 1.14 (1.06,1.26) | 1.21 (1.08,1.3) | 1.13 (1.05,1.25) | 6.26 | 0.1 |
| FALFF-lLTC | 1.11 (1.03,1.22) | 1.12 (1.03,1.18) | 1.14 (1.02,1.25) | 1.12 (1.01,1.2) | 1.1 (1.05,1.24) | 1.8 | 0.614 |
| FALFF-rLTC | 1.13 (1.04,1.24) | 1.13 (1.04,1.22) | 1.12 (1.03,1.27) | 1.12 (1.05,1.23) | 1.14 (0.99,1.21) | 0.48 | 0.923 |
| FALFF-lPHF | 0.84 (0.8,0.89) | 0.84 (0.81,0.88) | 0.85 (0.79,0.89) | 0.84 (0.8,0.89) | 0.84 (0.79,0.88) | 0.58 | 0.902 |
| FALFF-rPHF | 0.84 (0.8,0.88) | 0.85 (0.81,0.89) | 0.83 (0.8,0.88) | 0.85 (0.8,0.88) | 0.83 (0.8,0.87) | 4.16 | 0.244 |
| **Mattsson stage** | **Negative** | **Early** | **Intermediate** | **Late** | **UND** |  |  |
| FALFF-amPFC | 1.22 (1.1,1.32) | 1.21 (1.13,1.33) | 1.22 (1.12,1.33) | 1.24 (1.13,1.39) | 1.21 (1.1,1.36) | 3.79 | 0.285 |
| FALFF-PRC | 1.27 (1.17,1.4) | 1.29 (1.19,1.38) | 1.26 (1.15,1.36) | 1.24 (1.13,1.32) | 1.32 (1.24,1.39) | 11.58 | 0.009 |
| FALFF-lIPC | 1.24 (1.13,1.36) | 1.24 (1.13,1.36) | 1.24 (1.14,1.34) | 1.22 (1.12,1.34) | 1.27 (1.19,1.44) | 1.94 | 0.585 |
| FALFF-rIPC | 1.4 (1.26,1.55) | 1.48 (1.34,1.6) | 1.42 (1.24,1.56) | 1.36 (1.21,1.52) | 1.44 (1.29,1.56) | 13.89 | 0.003 |
| FALFF-vmPFC | 0.9 (0.84,0.97) | 0.92 (0.87,0.99) | 0.9 (0.85,0.97) | 0.9 (0.85,0.97) | 0.86 (0.81,0.93) | 4.07 | 0.254 |
| FALFF-dmPFC | 1.16 (1.06,1.29) | 1.18 (1.09,1.28) | 1.15 (1.07,1.26) | 1.21 (1.08,1.3) | 1.13 (1.05,1.25) | 4.72 | 0.194 |
| FALFF-lLTC | 1.11 (1.03,1.22) | 1.12 (1.03,1.19) | 1.12 (1.02,1.23) | 1.12 (1.01,1.2) | 1.1 (1.05,1.24) | 0.31 | 0.959 |
| FALFF-rLTC | 1.13 (1.04,1.24) | 1.14 (1.04,1.24) | 1.13 (1.04,1.24) | 1.11 (1.04,1.23) | 1.14 (0.99,1.21) | 0.62 | 0.892 |
| FALFF-lPHF | 0.84 (0.8,0.89) | 0.84 (0.81,0.88) | 0.85 (0.8,0.89) | 0.84 (0.8,0.89) | 0.84 (0.79,0.88) | 0.87 | 0.832 |
| FALFF-rPHF | 0.84 (0.8,0.88) | 0.85 (0.8,0.88) | 0.84 (0.8,0.89) | 0.85 (0.8,0.88) | 0.83 (0.8,0.87) | 2.61 | 0.456 |

Note: UND indicates "undetermined," and this group is not included in the statistical analysis. amPFC, anterior medial prefrontal cortex; PRC, precuneus; lIPC, Left intraparietal cortex; rIPC, Right intraparietal cortex; vmPFC, ventromedial prefrontal cortex; dmPFC, dorsomedial prefrontal cortex; lLTC, Left lateral temporal cortex; rLTC, Right lateral temporal cortex; lPHF, Left paraHipp formation; rPHF, Right paraHipp formation.

**Supplementary Table 5 Posterior connections of Default Mode Network in different Aβ-PET stages**

|  | **Stage** | | | | | **Statistic** | **P** |
| --- | --- | --- | --- | --- | --- | --- | --- |
| **Villeneuve stage** | **Negative** | **Regional** | **Widespread** |  |  |  |  |
| PRC-amPFC | 0.77 (0.53,0.98) | 0.79 (0.56,1.03) | 0.8 (0.56,1) |  |  | 2.15 | 0.342 |
| PRC-lIPC | 0.74 (0.45,0.94) | 0.74 (0.52,0.99) | 0.73 (0.51,0.89) |  |  | 3.14 | 0.208 |
| PRC-rIPC | 0.82 (0.61,1.05) | 0.83 (0.6,1.09) | 0.8 (0.56,1.05) |  |  | 1.34 | 0.511 |
| PRC-vmPFC | 0.2 (0.05,0.36) | 0.17 (0.01,0.33) | 0.23 (0.06,0.36) |  |  | 5.04 | 0.08 |
| PRC-dmPFC | 0.58 (0.4,0.78) | 0.6 (0.42,0.79) | 0.59 (0.38,0.8) |  |  | 0.81 | 0.668 |
| PRC-lLTC | 0.03 (-0.15,0.23) | 0 (-0.17,0.18) | -0.02 (-0.2,0.17) |  |  | 4.64 | 0.098 |
| PRC-rLTC | 0.06 (-0.15,0.24) | 0.02 (-0.16,0.19) | 0.01 (-0.14,0.19) |  |  | 3.15 | 0.207 |
| PRC-lPHF | 0.28 (0.15,0.43) | 0.27 (0.1,0.41) | 0.2 (0.08,0.35) |  |  | 14.97 | < .001 |
| PRC-rPHF | 0.21 (0.07,0.35) | 0.17 (0.03,0.34) | 0.16 (0.02,0.3) |  |  | 6.76 | 0.034 |
| **Grothe stage** | **Stage 0** | **Stage 1+2** | **Stage 3** | **Stage 4** | **UND** |  |  |
| PRC-amPFC | 0.77 (0.53,0.98) | 0.8 (0.56,1) | 0.74 (0.5,1.02) | 0.82 (0.56,1.01) | 0.82 (0.58,1.11) | 2.65 | 0.45 |
| PRC-lIPC | 0.74 (0.45,0.94) | 0.77 (0.52,1.03) | 0.69 (0.54,0.89) | 0.74 (0.5,0.92) | 0.79 (0.54,1.07) | 4.73 | 0.193 |
| PRC-rIPC | 0.82 (0.61,1.05) | 0.86 (0.64,1.09) | 0.81 (0.59,1.04) | 0.8 (0.54,1.05) | 0.8 (0.6,1.17) | 2.09 | 0.555 |
| PRC-vmPFC | 0.2 (0.05,0.36) | 0.15 (0.01,0.32) | 0.21 (0.01,0.38) | 0.21 (0.04,0.35) | 0.17 (0.03,0.29) | 3.22 | 0.36 |
| PRC-dmPFC | 0.58 (0.4,0.78) | 0.61 (0.44,0.79) | 0.6 (0.34,0.78) | 0.57 (0.37,0.81) | 0.63 (0.51,0.9) | 2.83 | 0.419 |
| PRC-lLTC | 0.03 (-0.15,0.23) | -0.01 (-0.16,0.17) | 0.02 (-0.16,0.18) | -0.01 (-0.2,0.17) | -0.01 (-0.23,0.14) | 4.1 | 0.251 |
| PRC-rLTC | 0.06 (-0.15,0.24) | 0 (-0.16,0.17) | 0.02 (-0.16,0.23) | 0.02 (-0.14,0.2) | 0.1 (-0.14,0.22) | 3.96 | 0.265 |
| PRC-lPHF | 0.28 (0.15,0.43) | 0.27 (0.1,0.42) | 0.24 (0.11,0.38) | 0.2 (0.08,0.34) | 0.29 (0.13,0.48) | 17.31 | < .001 |
| PRC-rPHF | 0.21 (0.07,0.35) | 0.17 (0.04,0.33) | 0.18 (0.04,0.35) | 0.16 (0.02,0.3) | 0.19 (-0.02,0.32) | 6.68 | 0.083 |
| **Mattsson stage** | **Negative** | **Early** | **Intermediate** | **Late** | **UND** |  |  |
| PRC-amPFC | 0.77 (0.53,0.98) | 0.81 (0.57,1) | 0.76 (0.52,1.01) | 0.81 (0.56,1.01) | 0.82 (0.58,1.11) | 2.5 | 0.476 |
| PRC-lIPC | 0.74 (0.45,0.94) | 0.79 (0.59,1.07) | 0.71 (0.51,0.89) | 0.73 (0.5,0.89) | 0.79 (0.54,1.07) | 8.36 | 0.039 |
| PRC-rIPC | 0.82 (0.61,1.05) | 0.85 (0.68,1.09) | 0.83 (0.57,1.05) | 0.79 (0.54,1.04) | 0.8 (0.6,1.17) | 2.94 | 0.402 |
| PRC-vmPFC | 0.2 (0.05,0.36) | 0.17 (0.03,0.33) | 0.18 (-0.01,0.35) | 0.21 (0.05,0.35) | 0.17 (0.03,0.29) | 2.14 | 0.544 |
| PRC-dmPFC | 0.58 (0.4,0.78) | 0.61 (0.46,0.78) | 0.6 (0.37,0.79) | 0.59 (0.37,0.81) | 0.63 (0.51,0.9) | 1.53 | 0.675 |
| PRC-lLTC | 0.03 (-0.15,0.23) | 0.02 (-0.17,0.18) | 0 (-0.15,0.18) | -0.01 (-0.2,0.17) | -0.01 (-0.23,0.14) | 4.28 | 0.233 |
| PRC-rLTC | 0.06 (-0.15,0.24) | 0.02 (-0.13,0.18) | 0.01 (-0.15,0.19) | 0.01 (-0.15,0.19) | 0.1 (-0.14,0.22) | 3.5 | 0.321 |
| PRC-lPHF | 0.28 (0.15,0.43) | 0.28 (0.09,0.41) | 0.24 (0.11,0.38) | 0.2 (0.08,0.35) | 0.29 (0.13,0.48) | 16.58 | < .001 |
| PRC-rPHF | 0.21 (0.07,0.35) | 0.19 (0.05,0.34) | 0.17 (0.02,0.34) | 0.16 (0.02,0.3) | 0.19 (-0.02,0.32) | 7.75 | 0.051 |

Note: UND indicates "undetermined," and this group is not included in the statistical analysis. amPFC, anterior medial prefrontal cortex; PRC, precuneus; lIPC, Left intraparietal cortex; rIPC, Right intraparietal cortex; vmPFC, ventromedial prefrontal cortex; dmPFC, dorsomedial prefrontal cortex; lLTC, Left lateral temporal cortex; rLTC, Right lateral temporal cortex; lPHF, Left paraHipp formation; rPHF, Right paraHipp formation.

**Supplementary Table 6 Volume of hippocampal subfields in Villeneuve stage**

|  | **Stage** | | | **Statistic** | **P** |
| --- | --- | --- | --- | --- | --- |
|  | **Negative** | **Regional** | **Widespread** |  |  |
| Left-Subic-body | 234.95 (216.98,254.42) | 233.11 (209.91,252.56) | 194.93 (161.66,227.14) | 76.36 | < .001 |
| Left-CA1-body | 109.19 (96.34,121.19) | 107.39 (93.86,122.39) | 96.82 (82.38,107.98) | 32.59 | < .001 |
| Left-Subic-head | 177.32 (158.37,194.4) | 175.63 (154.67,194.83) | 145.81 (121.23,172.51) | 59.66 | < .001 |
| Left-Hipp-fissure | 153.35 (135.56,170.34) | 153.98 (134.77,172.98) | 149.71 (136.69,171.68) | 0.2 | 0.904 |
| Left-preSubic-head | 129.25 (118.86,141.2) | 130.18 (115.45,142.89) | 110.4 (93.51,127.66) | 59.01 | < .001 |
| Left-CA1-head | 473.46 (435.82,514.96) | 473.09 (428.24,527.19) | 415.15 (352.63,473.33) | 53.74 | < .001 |
| Left-preSubic-body | 160.43 (140.15,178.38) | 158.21 (135.22,180.47) | 128.31 (103.87,152.63) | 69.47 | < .001 |
| Left-paraSubic | 62.36 (54.97,70.47) | 64.06 (55.7,71.56) | 58.01 (48.57,67.7) | 11.71 | 0.003 |
| Left-ML-HP-head | 302.8 (280.33,326.98) | 305.52 (271.7,336.86) | 256.32 (218.39,299.28) | 65.01 | < .001 |
| Left-ML-HP-body | 205.92 (191.74,220.8) | 207.47 (186.15,221.55) | 168.47 (144.11,200.01) | 78.88 | < .001 |
| Left-GC-ML-DG-head | 138.3 (126.79,152.06) | 141.46 (124.44,154.04) | 120.67 (103.08,136.11) | 59.29 | < .001 |
| Left-CA3-body | 79.75 (70.09,89.62) | 79.3 (67.08,89.34) | 69.25 (59.96,84.65) | 22.46 | < .001 |
| Left-GC-ML-DG-body | 125.19 (116.15,134.05) | 124.94 (111.86,132.94) | 106.66 (90.67,124.49) | 68.96 | < .001 |
| Left-CA4-head | 117.6 (107.91,128.05) | 119.6 (107.47,129.45) | 103.75 (88.43,117.45) | 60.1 | < .001 |
| Left-CA4-body | 112.59 (104.46,121.29) | 112.34 (100.84,119.71) | 95.65 (82.72,112.32) | 66.01 | < .001 |
| Left-fimbria | 72.02 (61.36,85.31) | 70.02 (56.14,84.71) | 58.29 (38.26,71.62) | 41.54 | < .001 |
| Left-CA3-head | 108.73 (98.48,119.99) | 109.53 (97.06,122.09) | 97.14 (83.73,110.43) | 41.62 | < .001 |
| Left-HATA | 48.21 (43.02,53.76) | 48.43 (41.78,54.35) | 42.2 (35.16,48.27) | 43.16 | < .001 |
| Right-Subic-body | 237.28 (217.72,256.52) | 236.31 (212.48,254.87) | 193.06 (159.13,235.96) | 74.55 | < .001 |
| Right-CA1-body | 123.23 (110.52,137.64) | 124.84 (109.11,138.57) | 107.56 (95.39,125.7) | 47.03 | < .001 |
| Right-Subic-head | 179.7 (161.05,199.06) | 179.82 (156.11,200.2) | 147.72 (124.1,177.08) | 70.3 | < .001 |
| Right-Hipp-fissure | 163.52 (144.62,188.64) | 165.85 (147.77,190.5) | 168.33 (154.1,191.7) | 3.62 | 0.163 |
| Right-preSubic-head | 127.19 (115.71,138.18) | 128.85 (113.37,139.88) | 107.02 (88.63,126.44) | 66.18 | < .001 |
| Right-CA1-head | 510.1 (462.81,555.77) | 513.04 (457.49,556.14) | 443.52 (385.73,497.47) | 60.95 | < .001 |
| Right-preSubic-body | 143.79 (127.07,163.25) | 144.29 (127.1,163.46) | 118.07 (98.96,143.63) | 63.02 | < .001 |
| Right-paraSubic | 57.4 (49.74,65.43) | 58.3 (50.63,67.78) | 50.77 (41.83,62.88) | 23.55 | < .001 |
| Right-ML-HP-head | 318.99 (292.98,343.19) | 320.53 (288.09,343.99) | 269.91 (229.16,313.51) | 71.14 | < .001 |
| Right-ML-HP-body | 219.11 (202.89,235.23) | 224.28 (199.06,238.89) | 177.58 (150.26,217.71) | 78.42 | < .001 |
| Right-GC-ML-DG-head | 147.05 (134.08,160.3) | 149.94 (134.36,162.26) | 130.59 (109.06,146.73) | 53.87 | < .001 |
| Right-CA3-body | 93.21 (82.25,106.68) | 95.32 (82.26,106.81) | 81.72 (70.22,96.5) | 36.32 | < .001 |
| Right-GC-ML-DG-body | 129.5 (120.52,141.87) | 131.26 (119.09,141.9) | 110.25 (96.81,131.71) | 63.94 | < .001 |
| Right-CA4-head | 124.17 (114.38,133.71) | 126.88 (114.85,137.4) | 112.37 (94.07,127.3) | 52.24 | < .001 |
| Right-CA4-body | 117.41 (108.7,128.37) | 119.4 (107.79,129.83) | 100.27 (89.33,120.05) | 59.69 | < .001 |
| Right-fimbria | 63.65 (50.63,76.58) | 60.19 (47.35,74.38) | 48.06 (33.86,64.25) | 41.92 | < .001 |
| Right-CA3-head | 115.26 (104.18,127.73) | 118.56 (104.25,130.67) | 104.47 (89.28,116.1) | 40.39 | < .001 |
| Right-HATA | 49.33 (44.17,54.87) | 50.49 (43.05,55.46) | 43.47 (35.36,48.59) | 54.25 | < .001 |

**Supplementary Table 7 Volume of hippocampal subfields in Grothe stage**

|  | **Stage** | | | | | **Statistic** | **P** |
| --- | --- | --- | --- | --- | --- | --- | --- |
|  | **Stage 0** | **Stage 1+2** | **Stage 3** | **Stage 4** | **UND** |  |  |
| Left-Subic-body | 234.95 (216.98,254.42) | 240.99 (219.25,255) | 219.48 (196.52,244.38) | 187.16 (159.45,227.06) | 227.02 (195.27,241.11) | 90.26 | < .001 |
| Left-CA1-body | 109.19 (96.34,121.19) | 111.13 (96.57,124.71) | 105.71 (89.75,125.44) | 96.02 (81.6,106.85) | 103.01 (93.67,117.64) | 44.48 | < .001 |
| Left-Subic-head | 177.32 (158.37,194.4) | 182.96 (164.17,204.51) | 165.93 (142.58,184.49) | 142.91 (120.24,170.16) | 168.87 (151.94,179.79) | 78.93 | < .001 |
| Left-Hipp-fissure | 153.35 (135.56,170.34) | 151.16 (134.04,169.22) | 163.27 (136.63,178.78) | 147.73 (136.3,170.07) | 155.27 (140.63,168.41) | 5.74 | 0.125 |
| Left-preSubic-head | 129.25 (118.86,141.2) | 136.05 (123.15,147.18) | 123.31 (107.28,134.6) | 109.42 (92.81,127.58) | 120.04 (109.22,134.04) | 79.91 | < .001 |
| Left-CA1-head | 473.46 (435.82,514.96) | 487.45 (447.3,539.84) | 451.56 (407.41,502.9) | 400.48 (351.3,467.66) | 443.57 (388.21,481.81) | 80.79 | < .001 |
| Left-preSubic-body | 160.43 (140.15,178.38) | 163.54 (146.61,186.08) | 148.85 (124.5,167.66) | 127.9 (103.02,154.6) | 147.34 (129.37,165.92) | 80.8 | < .001 |
| Left-paraSubic | 62.36 (54.97,70.47) | 65.53 (57.13,72.78) | 62.3 (54.1,71.5) | 57.73 (48.63,67.54) | 63.32 (51.48,68.54) | 14.98 | 0.002 |
| Left-ML-HP-head | 302.8 (280.33,326.98) | 317.89 (290.96,343.66) | 288.26 (261.21,317.66) | 249.76 (216.82,293.49) | 276.23 (252.04,310.81) | 94.59 | < .001 |
| Left-ML-HP-body | 205.92 (191.74,220.8) | 212.17 (200.04,223.93) | 197.18 (168.05,217.6) | 166.72 (141.29,198.14) | 195.7 (169.35,209.9) | 102.57 | < .001 |
| Left-GC-ML-DG-head | 138.3 (126.79,152.06) | 146.73 (134.38,156.7) | 133.49 (117.1,148) | 118.14 (100.87,133.28) | 126.99 (113.52,141.82) | 88.14 | < .001 |
| Left-CA3-body | 79.75 (70.09,89.62) | 80.85 (68.66,89.9) | 77.89 (66.66,93.23) | 68.54 (59.19,81.71) | 71.02 (62.74,88.31) | 31.1 | < .001 |
| Left-GC-ML-DG-body | 125.19 (116.15,134.05) | 128.3 (120.16,133.67) | 121.24 (106.43,130.47) | 102.65 (87.75,120.27) | 116.5 (102.61,130.59) | 95.64 | < .001 |
| Left-CA4-head | 117.6 (107.91,128.05) | 123.1 (113.75,131.9) | 113.07 (100.7,125.95) | 101.39 (87.8,114.95) | 109.1 (97.43,120.41) | 84.47 | < .001 |
| Left-CA4-body | 112.59 (104.46,121.29) | 114.73 (108.25,121.15) | 108.55 (96.1,118.86) | 93.51 (80.2,109.26) | 102.53 (94.87,117.38) | 92.16 | < .001 |
| Left-fimbria | 72.02 (61.36,85.31) | 74.94 (60.62,90.27) | 63.06 (49.03,78.07) | 57.59 (38.32,71.6) | 66.45 (54.47,78.92) | 54.6 | < .001 |
| Left-CA3-head | 108.73 (98.48,119.99) | 112.41 (103.28,126.11) | 108.06 (91.66,116.08) | 95.91 (81.8,108.03) | 103.04 (92.58,110.22) | 61.2 | < .001 |
| Left-HATA | 48.21 (43.02,53.76) | 49.76 (46.18,56.42) | 46.84 (37.75,53.19) | 42.02 (34.59,47.46) | 45.19 (36.38,49.36) | 63.87 | < .001 |
| Right-Subic-body | 237.28 (217.72,256.52) | 240.87 (224.97,260.91) | 227.28 (202.76,247.22) | 189.35 (156.26,235.52) | 223.54 (197.11,240.29) | 88.34 | < .001 |
| Right-CA1-body | 123.23 (110.52,137.64) | 128.09 (114.53,142.37) | 117.46 (107.17,135.46) | 106.75 (94.11,123.97) | 119.59 (95.42,134.95) | 64.76 | < .001 |
| Right-Subic-head | 179.7 (161.05,199.06) | 187.49 (170.59,208.93) | 170.79 (146.25,193.46) | 146.77 (121.99,172.28) | 170.77 (149.06,188.24) | 90.67 | < .001 |
| Right-Hipp-fissure | 163.52 (144.62,188.64) | 164.38 (147.79,185.44) | 172.67 (152.03,194.16) | 166.85 (154.25,191.65) | 160.3 (143.78,192.22) | 6.46 | 0.091 |
| Right-preSubic-head | 127.19 (115.71,138.18) | 132.54 (119.42,142.87) | 119.32 (106.47,136.82) | 106.12 (88.01,124.91) | 118.19 (102.79,129.6) | 86.76 | < .001 |
| Right-CA1-head | 510.1 (462.81,555.77) | 531.59 (488.22,568.7) | 498.67 (420.37,538.49) | 440.74 (377.31,489.92) | 461.73 (428.52,513.44) | 83.73 | < .001 |
| Right-preSubic-body | 143.79 (127.07,163.25) | 147.36 (131.35,166.94) | 134.94 (115.74,158.3) | 117.99 (92.75,142.9) | 136.37 (122.49,154.09) | 68.57 | < .001 |
| Right-paraSubic | 57.4 (49.74,65.43) | 59.07 (52.34,69.53) | 56.3 (49.13,66.6) | 50.8 (40.93,62.86) | 53.32 (48.99,62.62) | 25.1 | < .001 |
| Right-ML-HP-head | 318.99 (292.98,343.19) | 335.99 (307.96,352.59) | 315.15 (264.97,331.05) | 267.06 (225.41,309.43) | 287.93 (268.93,318.02) | 98.52 | < .001 |
| Right-ML-HP-body | 219.11 (202.89,235.23) | 230.6 (213.04,242.79) | 212.75 (178.48,231.96) | 174.85 (146.73,213.52) | 196.49 (182.4,229.5) | 106.14 | < .001 |
| Right-GC-ML-DG-head | 147.05 (134.08,160.3) | 155.78 (140.5,165.8) | 143.08 (124.56,158.07) | 128.56 (106.93,144.68) | 135.07 (120.89,150.3) | 78.37 | < .001 |
| Right-CA3-body | 93.21 (82.25,106.68) | 98.29 (87.62,107.71) | 90.56 (78.79,107.05) | 81.44 (69.25,92.95) | 83.07 (72.73,96.47) | 50.71 | < .001 |
| Right-GC-ML-DG-body | 129.5 (120.52,141.87) | 135.53 (127.26,145.33) | 125.66 (111.13,137.39) | 107.53 (95.41,127.22) | 117.06 (107.91,127.86) | 92.65 | < .001 |
| Right-CA4-head | 124.17 (114.38,133.71) | 130.73 (119.08,139.74) | 122.54 (109.85,133.12) | 110.91 (93.24,126.19) | 117.47 (103.59,125.76) | 72.3 | < .001 |
| Right-CA4-body | 117.41 (108.7,128.37) | 122.19 (115.35,132.29) | 113.92 (100.17,127.38) | 98.86 (87.11,118.62) | 110.29 (97.55,118.68) | 86.47 | < .001 |
| Right-fimbria | 63.65 (50.63,76.58) | 61.91 (50.39,77.71) | 56.77 (43.58,67.74) | 47.67 (31.94,63.69) | 57.37 (37.78,72.95) | 51.3 | < .001 |
| Right-CA3-head | 115.26 (104.18,127.73) | 121.86 (110.27,132.28) | 113.76 (100.29,127.28) | 102.34 (88.52,116.1) | 107.17 (93.81,121.35) | 54.45 | < .001 |
| Right-HATA | 49.33 (44.17,54.87) | 52.78 (47.26,56.87) | 47.74 (38.56,53.4) | 42.75 (35.29,48.58) | 45.7 (40.75,50.52) | 67.8 | < .001 |

**Supplementary Table 8 Volume of hippocampal subfields in Mattsson stage**

|  | **Stage** | | | | | **Statistic** | **P** |
| --- | --- | --- | --- | --- | --- | --- | --- |
|  | **Negative** | **Early** | **Intermediate** | **Late** | **UND** |  |  |
| Left-Subic-body | 234.95 (216.98,254.42) | 241.03 (222.32,260.31) | 226.37 (204.3,250.05) | 189.96 (160.29,226.98) | 227.02 (195.27,241.11) | 86.59 | < .001 |
| Left-CA1-body | 109.19 (96.34,121.19) | 113.65 (97.84,126.32) | 106.04 (92.38,120.61) | 96.34 (82.1,107.26) | 103.01 (93.67,117.64) | 40.57 | < .001 |
| Left-Subic-head | 177.32 (158.37,194.4) | 184.67 (164.62,204.56) | 170.54 (147.15,191.65) | 145.06 (120.25,171.83) | 168.87 (151.94,179.79) | 70.29 | < .001 |
| Left-Hipp-fissure | 153.35 (135.56,170.34) | 154.35 (136.44,173.61) | 154.04 (131.85,173.09) | 147.77 (136.45,171.46) | 155.27 (140.63,168.41) | 0.65 | 0.885 |
| Left-preSubic-head | 129.25 (118.86,141.2) | 136.03 (121.81,147.73) | 127.87 (113.97,141.22) | 110.32 (92.84,127.55) | 120.04 (109.22,134.04) | 69.18 | < .001 |
| Left-CA1-head | 473.46 (435.82,514.96) | 493.48 (447.63,537.94) | 460.83 (422.23,525.93) | 402.8 (351.37,468.3) | 443.57 (388.21,481.81) | 70.49 | < .001 |
| Left-preSubic-body | 160.43 (140.15,178.38) | 162.42 (143.21,186.05) | 156.64 (130.71,175.25) | 127.94 (103.22,153.91) | 147.34 (129.37,165.92) | 73.71 | < .001 |
| Left-paraSubic | 62.36 (54.97,70.47) | 64.64 (57.01,71.71) | 63.86 (56.16,72.29) | 58.01 (48.68,67.67) | 63.32 (51.48,68.54) | 13.46 | 0.004 |
| Left-ML-HP-head | 302.8 (280.33,326.98) | 317.41 (291.08,344.2) | 296.52 (264.68,334.14) | 251.37 (217.66,296.34) | 276.23 (252.04,310.81) | 82.02 | < .001 |
| Left-ML-HP-body | 205.92 (191.74,220.8) | 212.51 (200.11,225.48) | 202.49 (178.32,220.03) | 167.3 (142.07,199.04) | 195.7 (169.35,209.9) | 94.14 | < .001 |
| Left-GC-ML-DG-head | 138.3 (126.79,152.06) | 144.64 (134.5,156.5) | 139.65 (120.17,153.13) | 119.05 (102.31,133.59) | 126.99 (113.52,141.82) | 77.59 | < .001 |
| Left-CA3-body | 79.75 (70.09,89.62) | 81.86 (70.38,90.59) | 76.15 (65.23,89.38) | 69 (59.72,83.1) | 71.02 (62.74,88.31) | 29.53 | < .001 |
| Left-GC-ML-DG-body | 125.19 (116.15,134.05) | 128.61 (119.94,133.97) | 124.28 (109,131.93) | 104.16 (89.05,123.04) | 116.5 (102.61,130.59) | 83.07 | < .001 |
| Left-CA4-head | 117.6 (107.91,128.05) | 122.21 (114.17,131.47) | 119.28 (104.06,129.38) | 102.2 (88.22,115.29) | 109.1 (97.43,120.41) | 76.11 | < .001 |
| Left-CA4-body | 112.59 (104.46,121.29) | 114.99 (108.24,121.72) | 111.06 (98.2,119.02) | 94.52 (81.73,110.6) | 102.53 (94.87,117.38) | 81.37 | < .001 |
| Left-fimbria | 72.02 (61.36,85.31) | 73.32 (58.97,87.3) | 66.01 (50.39,82.53) | 58.29 (38.39,71.57) | 66.45 (54.47,78.92) | 44.97 | < .001 |
| Left-CA3-head | 108.73 (98.48,119.99) | 112.69 (104.6,127.23) | 109.2 (93.88,120.53) | 96.1 (83.01,108.44) | 103.04 (92.58,110.22) | 57.32 | < .001 |
| Left-HATA | 48.21 (43.02,53.76) | 49.39 (46.2,56.38) | 48.21 (40.05,54.68) | 42.09 (33.99,47.62) | 45.19 (36.38,49.36) | 56.53 | < .001 |
| Right-Subic-body | 237.28 (217.72,256.52) | 243.27 (229.93,262.46) | 227.73 (206.38,249.12) | 192.36 (157.12,236.54) | 223.54 (197.11,240.29) | 88.67 | < .001 |
| Right-CA1-body | 123.23 (110.52,137.64) | 130.77 (117.1,143.6) | 119.11 (107.62,135.04) | 107.01 (94.31,124.73) | 119.59 (95.42,134.95) | 64.32 | < .001 |
| Right-Subic-head | 179.7 (161.05,199.06) | 192.02 (170.7,209.76) | 175.03 (148.1,195.03) | 147.72 (123.01,176.6) | 170.77 (149.06,188.24) | 82.25 | < .001 |
| Right-Hipp-fissure | 163.52 (144.62,188.64) | 166.26 (149.67,191.08) | 167.46 (146.82,187.75) | 167.51 (153.99,191.77) | 160.3 (143.78,192.22) | 4.2 | 0.241 |
| Right-preSubic-head | 127.19 (115.71,138.18) | 132.64 (119.95,142.18) | 125.25 (110.33,138.17) | 106.71 (88.27,126.21) | 118.19 (102.79,129.6) | 75.8 | < .001 |
| Right-CA1-head | 510.1 (462.81,555.77) | 538.78 (492.75,568.06) | 502.09 (442.38,543.71) | 441.44 (379.59,493.5) | 461.73 (428.52,513.44) | 77.82 | < .001 |
| Right-preSubic-body | 143.79 (127.07,163.25) | 146.51 (130.6,167.11) | 139.34 (122.43,161.42) | 118 (96.08,144.49) | 136.37 (122.49,154.09) | 64.44 | < .001 |
| Right-paraSubic | 57.4 (49.74,65.43) | 59.15 (52.35,69.15) | 57.5 (50.79,67.25) | 50.77 (41.23,63.14) | 53.32 (48.99,62.62) | 24.33 | < .001 |
| Right-ML-HP-head | 318.99 (292.98,343.19) | 336.89 (313.42,352.16) | 316.04 (279.58,337.62) | 267.54 (226.62,312.11) | 287.93 (268.93,318.02) | 88.74 | < .001 |
| Right-ML-HP-body | 219.11 (202.89,235.23) | 230.9 (218.28,248.31) | 216.71 (191.08,234.98) | 176.55 (147.05,216.87) | 196.49 (182.4,229.5) | 102.51 | < .001 |
| Right-GC-ML-DG-head | 147.05 (134.08,160.3) | 156.56 (143.13,165.94) | 145.77 (129.23,158.78) | 129.41 (108.42,146.54) | 135.07 (120.89,150.3) | 71.2 | < .001 |
| Right-CA3-body | 93.21 (82.25,106.68) | 99.86 (92.21,110.16) | 91.5 (79.83,104.39) | 81.44 (69.48,95.01) | 83.07 (72.73,96.47) | 53.67 | < .001 |
| Right-GC-ML-DG-body | 129.5 (120.52,141.87) | 136.1 (128.53,146.14) | 128.98 (114.53,140.46) | 108.09 (95.99,127.6) | 117.06 (107.91,127.86) | 84.1 | < .001 |
| Right-CA4-head | 124.17 (114.38,133.71) | 131.3 (121.71,139.73) | 122.72 (113.05,135.03) | 112.16 (93.44,127.04) | 117.47 (103.59,125.76) | 67.16 | < .001 |
| Right-CA4-body | 117.41 (108.7,128.37) | 123.54 (115.81,132.29) | 117.49 (103.63,128.21) | 99.38 (88.31,119.43) | 110.29 (97.55,118.68) | 80.63 | < .001 |
| Right-fimbria | 63.65 (50.63,76.58) | 61.08 (49.01,74.62) | 59.71 (46.35,72.24) | 47.83 (33.62,63.9) | 57.37 (37.78,72.95) | 43.79 | < .001 |
| Right-CA3-head | 115.26 (104.18,127.73) | 122.3 (111.48,133.93) | 117.21 (102.48,129.16) | 103.76 (89.06,116.1) | 107.17 (93.81,121.35) | 51.62 | < .001 |
| Right-HATA | 49.33 (44.17,54.87) | 52.1 (47.26,55.57) | 49.19 (40.86,55.88) | 43.03 (35.31,48.57) | 45.7 (40.75,50.52) | 61.74 | < .001 |

**Supplementary Table 9 Hippocampal volume at different Aβ-stages in cognitively unimpaired population**

|  | **Stage** | | |  |  | **Statistic** | **P** |
| --- | --- | --- | --- | --- | --- | --- | --- |
| **Villeneuve stage** | **Negative** | **Regional** | **Widespread** |  |  |  |  |
| Left-Hipp-head | 1573.61 (1476.44,1692.33) | 1646.51 (1521.01,1790.95) | 1640.39 (1454.87,1740.35) |  |  | 9.06 | 0.011 |
| Left- Hipp-body | 1120.43 (1049.21,1185.55) | 1148.21 (1078.76,1210.66) | 1115.3 (1063.46,1191.76) |  |  | 3.86 | 0.145 |
| Left-Hipp-tail | 541.25 (488.35,582.25) | 551.81 (495.21,594.14) | 540.11 (515.2,621.32) |  |  | 1.76 | 0.416 |
| Left- hippocampus | 3268.97 (3066.07,3406.87) | 3364.1 (3111.34,3545.8) | 3283.71 (2940.92,3649.27) |  |  | 7.23 | 0.027 |
| Right- Hipp-head | 1649.83 (1525.85,1760.84) | 1716.95 (1617.72,1822.68) | 1633.62 (1566.59,1787.14) |  |  | 12.22 | 0.002 |
| Right- Hipp-body | 1162.97 (1082.53,1212.3) | 1192 (1132.03,1260.71) | 1159.64 (1094.43,1239.17) |  |  | 9.34 | 0.009 |
| Right-Hipp-tail | 579.06 (527.1,614.05) | 593.55 (530.46,645.94) | 592.3 (547.49,608.3) |  |  | 4.69 | 0.096 |
| Right- hippocampus | 3363.43 (3181.29,3565.96) | 3509.7 (3287.86,3729.28) | 3354.35 (3242.19,3658.83) |  |  | 11.01 | 0.004 |
| **Grothe stage** | **Stage 0** | **Stage 1+2** | **Stage 3** | **Stage 4** | **UND** |  |  |
| Left- Hipp-head | 1573.61 (1476.44,1692.33) | 1673.98 (1541.95,1808.76) | 1616.55 (1485.92,1734.19) | 1663.02 (1443.95,1753.42) | 1588.45 (1536.1,1669.81) | 10.85 | 0.013 |
| Left- Hipp-body | 1120.43 (1049.21,1185.55) | 1152.09 (1092.24,1211.07) | 1115.3 (1026.74,1206.49) | 1143.57 (1083.73,1209.95) | 1137.69 (1071.93,1214.3) | 5.13 | 0.162 |
| Left-Hipp-tail | 541.25 (488.35,582.25) | 549.17 (504.94,591.16) | 548.16 (508.86,585.2) | 549.94 (521.06,625.95) | 572.03 (435.67,617.78) | 2.65 | 0.449 |
| Left- hippocampus | 3268.97 (3066.07,3406.87) | 3401.89 (3171.99,3564.88) | 3283.71 (2994.98,3522.05) | 3374.12 (2934.14,3655.97) | 3242.45 (3170.09,3373.29) | 9.24 | 0.026 |
| Right- Hipp-head | 1649.83 (1525.85,1760.84) | 1752.61 (1601.25,1844.04) | 1690.29 (1636.04,1765.64) | 1614.67 (1550.9,1790.74) | 1657.93 (1585.98,1700.53) | 14.37 | 0.002 |
| Right-Hipp-body | 1162.97 (1082.53,1212.3) | 1195.41 (1140.66,1260.71) | 1163.57 (1102.3,1238.99) | 1198.34 (1159.24,1253.93) | 1184.12 (1095.46,1220.41) | 10.61 | 0.014 |
| Right-Hipp-tail | 579.06 (527.1,614.05) | 594.19 (549.13,649.86) | 583.86 (514.77,616.83) | 599.09 (574.66,645.42) | 599.6 (542.86,659.61) | 6.26 | 0.1 |
| Right- hippocampus | 3363.43 (3181.29,3565.96) | 3539.49 (3297.64,3731.88) | 3406.09 (3234.81,3614.95) | 3440.66 (3318.74,3737.78) | 3382.02 (3244.52,3505.83) | 12.95 | 0.005 |
| **Mattsson Stage** | **Negative** | **Early** | **Intermediate** | **Late** | **UND** |  |  |
| Left-Hipp-head | 1573.61 (1476.44,1692.33) | 1633.69 (1521.01,1806.65) | 1669.29 (1525.07,1764.44) | 1620.85 (1453.44,1753.42) | 1588.45 (1536.1,1669.81) | 9.96 | 0.019 |
| Left-Hipp-body | 1120.43 (1049.21,1185.55) | 1146.81 (1096.34,1231.26) | 1153.72 (1061.37,1205.03) | 1113.75 (1050.9,1200.64) | 1137.69 (1071.93,1214.3) | 4.67 | 0.197 |
| Left-Hipp-tail | 541.25 (488.35,582.25) | 551.81 (512.87,591.16) | 548.96 (485.24,585.38) | 541.64 (511.25,621.89) | 572.03 (435.67,617.78) | 2.12 | 0.548 |
| Left-hippocampus | 3268.97 (3066.07,3406.87) | 3381.2 (3171.99,3555.44) | 3364.1 (3057.97,3531.55) | 3264.08 (2935.62,3655.97) | 3242.45 (3170.09,3373.29) | 8.19 | 0.042 |
| Right-Hipp-head | 1649.83 (1525.85,1760.84) | 1757.89 (1624.81,1832.65) | 1707.58 (1609.94,1826.53) | 1660.36 (1573.02,1790.74) | 1657.93 (1585.98,1700.53) | 13.39 | 0.004 |
| Right-Hipp-body | 1162.97 (1082.53,1212.3) | 1192 (1153.48,1272.29) | 1186.31 (1108.3,1247.72) | 1169.58 (1092.12,1240.6) | 1184.12 (1095.46,1220.41) | 10.85 | 0.013 |
| Right-Hipp-tail | 579.06 (527.1,614.05) | 593.55 (550.88,639.54) | 594.19 (511.38,643.76) | 590.84 (546.38,608.84) | 599.6 (542.86,659.61) | 4.98 | 0.173 |
| Right- hippocampus | 3363.43 (3181.29,3565.96) | 3532.36 (3315.38,3735.36) | 3496.05 (3243.55,3728.63) | 3364.35 (3255.43,3672.06) | 3382.02 (3244.52,3505.83) | 12.77 | 0.005 |

Note: cognitively unimpaired population includes NC and SCD. Hipp, Hippocampal.

**Supplementary Table 10 Hippocampal volume at different Aβ-stages in MCI**

|  | **Stage** | | |  |  | **Statistic** | **P** |
| --- | --- | --- | --- | --- | --- | --- | --- |
| **Villeneuve stage** | **Negative** | **Regional** | **Widespread** |  |  |  |  |
| Left-Hipp-head | 1549.76 (1439.53,1708.49) | 1549.29 (1368.82,1668.76) | 1524.31 (1357.06,1622.68) |  |  | 3.35 | 0.187 |
| Left- Hipp-body | 1094.06 (1032.74,1166.04) | 1092.64 (980.67,1158.17) | 1022.17 (941.48,1136.31) |  |  | 4.88 | 0.087 |
| Left-Hipp-tail | 522.74 (476.51,563.4) | 510.35 (465.44,553.25) | 494.79 (436.71,571.92) |  |  | 2.86 | 0.239 |
| Left- hippocampus | 3180.87 (2953.14,3397.28) | 3186.5 (2891.56,3360.76) | 3062.9 (2827.52,3358.57) |  |  | 4.31 | 0.116 |
| Right- Hipp-head | 1637.91 (1516.75,1779.77) | 1638.91 (1471.31,1753.66) | 1532.18 (1385.73,1641.07) |  |  | 8.21 | 0.016 |
| Right- Hipp-body | 1134.94 (1054.48,1216.46) | 1129.22 (1014.61,1200.46) | 1062.39 (918.33,1171.83) |  |  | 5.53 | 0.063 |
| Right-Hipp-tail | 550.28 (511.29,606.05) | 553.95 (504.21,606.66) | 537.78 (480.35,572.14) |  |  | 4.23 | 0.121 |
| Right- hippocampus | 3340.33 (3090.39,3564.05) | 3359.3 (2975.54,3489.79) | 3127.68 (2723.57,3387.12) |  |  | 7.3 | 0.026 |
| **Grothe stage** | **Stage 0** | **Stage 1+2** | **Stage 3** | **Stage 4** | **UND** |  |  |
| Left- Hipp-head | 1549.76 (1439.53,1708.49) | 1612.07 (1516.26,1747.53) | 1475.21 (1325.98,1617.53) | 1529.67 (1345.95,1615.07) | 1401.53 (1271.2,1453.9) | 12.22 | 0.007 |
| Left- Hipp-body | 1094.06 (1032.74,1166.04) | 1124.22 (1072.2,1171.06) | 1044.06 (956.65,1140.89) | 1009.42 (922.44,1133.35) | 1022.11 (880.52,1053.34) | 11.94 | 0.008 |
| Left-Hipp-tail | 522.74 (476.51,563.4) | 521.52 (478.3,565.03) | 505.75 (452.56,553.25) | 493.26 (416.05,574.21) | 507.24 (420.17,520.96) | 3.95 | 0.267 |
| Left- hippocampus | 3180.87 (2953.14,3397.28) | 3291.85 (3096.17,3397.46) | 2980.52 (2696.02,3323.11) | 3062.9 (2710.6,3330.6) | 2889.12 (2565.36,3048.48) | 12.25 | 0.007 |
| Right- Hipp-head | 1637.91 (1516.75,1779.77) | 1672.31 (1584.47,1775.39) | 1572.4 (1396.41,1676.58) | 1532.18 (1393.46,1633.67) | 1428.79 (1400.96,1509.66) | 15.58 | 0.001 |
| Right-Hipp-body | 1134.94 (1054.48,1216.46) | 1161.4 (1106.3,1230.59) | 1090.48 (943.44,1171.56) | 1062.39 (912.49,1169.8) | 981.84 (958.62,1057.9) | 14.05 | 0.003 |
| Right-Hipp-tail | 550.28 (511.29,606.05) | 571.4 (525.83,610.45) | 545.94 (479.14,591.88) | 537.78 (443.71,573.83) | 517.46 (460.32,542.57) | 7.42 | 0.06 |
| Right- hippocampus | 3340.33 (3090.39,3564.05) | 3441.45 (3272.06,3565.18) | 3184.8 (2826.33,3441.47) | 3188.85 (2712.62,3386.36) | 2990.23 (2869,3118.48) | 16.02 | 0.001 |
| **Mattsson Stage** | **Negative** | **Early** | **Intermediate** | **Late** | **UND** |  |  |
| Left-Hipp-head | 1549.76 (1439.53,1708.49) | 1619.05 (1516.26,1757.75) | 1522.29 (1359.2,1621.62) | 1526.99 (1315.16,1611.26) | 1401.53 (1271.2,1453.9) | 12.36 | 0.006 |
| Left-Hipp-body | 1094.06 (1032.74,1166.04) | 1124.22 (1072.2,1171.06) | 1063.79 (961.97,1153.13) | 1003.97 (927.86,1131.87) | 1022.11 (880.52,1053.34) | 10.79 | 0.013 |
| Left-Hipp-tail | 522.74 (476.51,563.4) | 525.89 (481.87,565.03) | 510.35 (450.19,556.05) | 494.02 (426.38,573.87) | 507.24 (420.17,520.96) | 3.76 | 0.289 |
| Left-hippocampus | 3180.87 (2953.14,3397.28) | 3293.96 (3110.36,3426.1) | 3058 (2827.52,3327.51) | 3017.17 (2577.14,3316.62) | 2889.12 (2565.36,3048.48) | 12.26 | 0.007 |
| Right-Hipp-head | 1637.91 (1516.75,1779.77) | 1693.19 (1611.36,1787.68) | 1585.92 (1456.48,1676.44) | 1521.37 (1380.13,1629.98) | 1428.79 (1400.96,1509.66) | 17.43 | < .001 |
| Right-Hipp-body | 1134.94 (1054.48,1216.46) | 1171.37 (1116.76,1240.71) | 1093.44 (951.77,1173.26) | 1087.14 (915.41,1168.79) | 981.84 (958.62,1057.9) | 16.6 | < .001 |
| Right-Hipp-tail | 550.28 (511.29,606.05) | 575.26 (527.52,615.99) | 547.29 (491.81,593.45) | 537.9 (449.71,573.41) | 517.46 (460.32,542.57) | 7.76 | 0.051 |
| Right- hippocampus | 3340.33 (3090.39,3564.05) | 3462.13 (3346.53,3589.1) | 3262.24 (2896.73,3442.11) | 3115.76 (2718.09,3385.98) | 2990.23 (2869,3118.48) | 18.33 | < .001 |

Note: Hipp, Hippocampal.

**Supplementary Table 11 Correlation between Aβ deposition and function in the Villeneuve stage**

| **Villeneuve Stage** | **Neuropsychological Test** | **Correlation** | | **P** | **Adjusted P** |
| --- | --- | --- | --- | --- | --- |
| **Negative** |  |  |  | |  |
|  | ADL | -0.05 | 0.308 | | 0.659 |
|  | FAQ | -0.04 | 0.406 | | 0.762 |
|  | MoCA-B | 0.06 | 0.167 | | 0.659 |
|  | AVLT long delay recall | 0.02 | 0.607 | | 0.843 |
|  | AVLT cue recall | -0.05 | 0.232 | | 0.659 |
|  | AVLT recognition | -0.02 | 0.65 | | 0.843 |
|  | STT-A | -0.08 | 0.064 | | 0.563 |
|  | STT-B | -0.03 | 0.574 | | 0.843 |
|  | BVMT long delay recall | -0.01 | 0.787 | | 0.843 |
|  | BVMT recognition | 0.08 | 0.075 | | 0.563 |
|  | BNT | 0.06 | 0.187 | | 0.659 |
|  | AFT | 0.05 | 0.272 | | 0.659 |
|  | CaST | -0.02 | 0.715 | | 0.843 |
|  | JLO | -0.02 | 0.737 | | 0.843 |
|  | ST | -0.01 | 0.85 | | 0.85 |
| **Regional** |  |  |  | |  |
|  | ADL | 0.11 | 0.055 | | 0.077 |
|  | FAQ | 0.11 | 0.064 | | 0.08 |
|  | MoCA-B | -0.22 | <.001 | | <.001 |
|  | AVLT long delay recall | -0.24 | <.001 | | <.001 |
|  | AVLT cue recall | -0.23 | <.001 | | <.001 |
|  | AVLT recognition | -0.25 | <.001 | | <.001 |
|  | STT-A | 0.11 | 0.056 | | 0.077 |
|  | STT-B | 0.19 | 0.002 | | 0.003 |
|  | BVMT long delay recall | -0.3 | <.001 | | <.001 |
|  | BVMT recognition | -0.26 | <.001 | | <.001 |
|  | BNT | -0.07 | 0.259 | | 0.299 |
|  | AFT | -0.2 | <.001 | | 0.001 |
|  | CaST | -0.18 | 0.004 | | 0.006 |
|  | JLO | -0.02 | 0.683 | | 0.683 |
|  | ST | -0.04 | 0.488 | | 0.523 |
| **Widespread** |  |  |  | |  |
|  | ADL | 0.21 | 0.006 | | 0.013 |
|  | FAQ | 0.29 | <.001 | | <.001 |
|  | MoCA-B | -0.21 | 0.007 | | 0.013 |
|  | AVLT long delay recall | -0.39 | <.001 | | <.001 |
|  | AVLT cue recall | -0.36 | <.001 | | <.001 |
|  | AVLT recognition | -0.19 | 0.047 | | 0.059 |
|  | STT-A | 0.24 | 0.013 | | 0.019 |
|  | STT-B | 0.22 | 0.025 | | 0.033 |
|  | BVMT long delay recall | -0.35 | <.001 | | 0.001 |
|  | BVMT recognition | -0.37 | <.001 | | <.001 |
|  | BNT | -0.06 | 0.574 | | 0.615 |
|  | AFT | -0.2 | 0.009 | | 0.015 |
|  | CaST | -0.48 | <.001 | | <.001 |
|  | JLO | -0.03 | 0.78 | | 0.78 |
|  | ST | -0.07 | 0.471 | | 0.544 |

Note: Corrected for age, sex, and years of education. ADL, Activity of Daily Living; FAQ, Functional Activities Questionnaire; MoCA-B, Montreal Cognitive Assessment-basic; AVLT, Auditory Verbal Learning Test; BVMT, Brief Visuospatial Memory Test; AFT, Animal-Verbal Fluency Test; BNT, Boston Naming Test; STT, Shape Trail Test; CaST, Category Switching Test; JOL, Judgement of Line Orientation. ST, Silhouettes Test.

**Supplementary Table 12 Correlation between Aβ deposition and function in the Grothe stage**

| **Grothe stage** | **Neuropsychological Test** | **Correlation** | **P** | **Adjusted P** |
| --- | --- | --- | --- | --- |
| **Stage 0** |  |  |  |  |
|  | ADL | -0.05 | 0.308 | 0.659 |
|  | FAQ | -0.04 | 0.406 | 0.762 |
|  | MoCA-B | 0.06 | 0.167 | 0.659 |
|  | AVLT long delay recall | 0.02 | 0.607 | 0.843 |
|  | AVLT cue recall | -0.05 | 0.232 | 0.659 |
|  | AVLT recognition | -0.02 | 0.650 | 0.843 |
|  | STT-A | -0.08 | 0.064 | 0.563 |
|  | STT-B | -0.03 | 0.574 | 0.843 |
|  | BVMT long delay recall | -0.01 | 0.787 | 0.843 |
|  | BVMT recognition | 0.08 | 0.075 | 0.563 |
|  | BNT | 0.06 | 0.187 | 0.659 |
|  | AFT | 0.05 | 0.272 | 0.659 |
|  | CaST | -0.02 | 0.715 | 0.843 |
|  | JLO | -0.02 | 0.737 | 0.843 |
|  | ST | -0.01 | 0.850 | 0.850 |
| **Stage 1+2** |  |  |  |  |
|  | ADL | -0.08 | 0.313 | 0.391 |
|  | FAQ | -0.10 | 0.208 | 0.300 |
|  | MoCA-B | -0.10 | 0.195 | 0.300 |
|  | AVLT long delay recall | -0.03 | 0.690 | 0.740 |
|  | AVLT cue recall | -0.05 | 0.533 | 0.616 |
|  | AVLT recognition | -0.19 | 0.021 | 0.160 |
|  | STT-A | 0.13 | 0.108 | 0.300 |
|  | STT-B | 0.11 | 0.196 | 0.300 |
|  | BVMT long delay recall | -0.12 | 0.142 | 0.300 |
|  | BVMT recognition | -0.14 | 0.085 | 0.300 |
|  | BNT | 0.02 | 0.812 | 0.812 |
|  | AFT | -0.20 | 0.012 | 0.160 |
|  | CaST | -0.11 | 0.220 | 0.300 |
|  | JLO | 0.13 | 0.111 | 0.300 |
|  | ST | 0.12 | 0.135 | 0.300 |
| **Stage 3** |  |  |  |  |
|  | ADL | 0.03 | 0.768 | 0.768 |
|  | FAQ | 0.08 | 0.414 | 0.478 |
|  | MoCA-B | -0.24 | 0.010 | 0.036 |
|  | AVLT long delay recall | -0.27 | 0.004 | 0.020 |
|  | AVLT cue recall | -0.21 | 0.027 | 0.081 |
|  | AVLT recognition | -0.10 | 0.300 | 0.409 |
|  | STT-A | 0.06 | 0.559 | 0.599 |
|  | STT-B | 0.18 | 0.063 | 0.148 |
|  | BVMT long delay recall | -0.35 | 0.000 | 0.004 |
|  | BVMT recognition | -0.31 | 0.001 | 0.009 |
|  | BNT | -0.09 | 0.356 | 0.445 |
|  | AFT | -0.14 | 0.132 | 0.199 |
|  | CaST | -0.15 | 0.127 | 0.199 |
|  | JLO | -0.18 | 0.069 | 0.148 |
|  | ST | -0.17 | 0.082 | 0.154 |
| **Stage 4** |  |  |  |  |
|  | ADL | 0.16 | 0.048 | 0.091 |
|  | FAQ | 0.22 | 0.005 | 0.019 |
|  | MoCA-B | -0.10 | 0.214 | 0.278 |
|  | AVLT long delay recall | -0.33 | 0.001 | 0.010 |
|  | AVLT cue recall | -0.28 | 0.008 | 0.020 |
|  | AVLT recognition | -0.13 | 0.223 | 0.278 |
|  | STT-A | 0.22 | 0.033 | 0.071 |
|  | STT-B | 0.19 | 0.074 | 0.123 |
|  | BVMT long delay recall | -0.31 | 0.004 | 0.018 |
|  | BVMT recognition | -0.29 | 0.006 | 0.019 |
|  | BNT | -0.01 | 0.922 | 0.922 |
|  | AFT | -0.13 | 0.107 | 0.161 |
|  | CaST | -0.44 | 0.000 | 0.001 |
|  | JLO | 0.01 | 0.916 | 0.922 |
|  | ST | -0.02 | 0.822 | 0.922 |

Note: Corrected for age, sex, and years of education. ADL, Activity of Daily Living; FAQ, Functional Activities Questionnaire; MoCA-B, Montreal Cognitive Assessment-basic; AVLT, Auditory Verbal Learning Test; BVMT, Brief Visuospatial Memory Test; AFT, Animal-Verbal Fluency Test; BNT, Boston Naming Test; STT, Shape Trail Test; CaST, Category Switching Test; JOL, Judgement of Line Orientation. ST, Silhouettes Test.

**Supplementary Table 13 Correlation between Aβ deposition and function in the Mattsson stage**

| **Mattsson stage** | **Neuropsychological Test** | **Correlation** | **P** | **Adjusted P** |
| --- | --- | --- | --- | --- |
| **Negative** |  |  |  |  |
|  | ADL | -0.05 | 0.308 | 0.659 |
|  | FAQ | -0.04 | 0.406 | 0.762 |
|  | MoCA-B | 0.06 | 0.167 | 0.659 |
|  | AVLT long delay recall | 0.02 | 0.607 | 0.843 |
|  | AVLT cue recall | -0.05 | 0.232 | 0.659 |
|  | AVLT recognition | -0.02 | 0.650 | 0.843 |
|  | STT-A | -0.08 | 0.064 | 0.563 |
|  | STT-B | -0.03 | 0.574 | 0.843 |
|  | BVMT long delay recall | -0.01 | 0.787 | 0.843 |
|  | BVMT recognition | 0.08 | 0.075 | 0.563 |
|  | BNT | 0.06 | 0.187 | 0.659 |
|  | AFT | 0.05 | 0.272 | 0.659 |
|  | CaST | -0.02 | 0.715 | 0.843 |
|  | JLO | -0.02 | 0.737 | 0.843 |
|  | ST | -0.01 | 0.850 | 0.850 |
| **Early** |  |  |  |  |
|  | ADL | -0.14 | 0.143 | 0.267 |
|  | FAQ | -0.06 | 0.525 | 0.606 |
|  | MoCA-B | -0.17 | 0.075 | 0.252 |
|  | AVLT long delay recall | -0.02 | 0.860 | 0.860 |
|  | AVLT cue recall | -0.09 | 0.376 | 0.512 |
|  | AVLT recognition | -0.15 | 0.120 | 0.263 |
|  | STT-A | 0.17 | 0.084 | 0.252 |
|  | STT-B | 0.15 | 0.123 | 0.263 |
|  | BVMT long delay recall | -0.14 | 0.167 | 0.279 |
|  | BVMT recognition | -0.24 | 0.013 | 0.190 |
|  | BNT | -0.04 | 0.654 | 0.700 |
|  | AFT | -0.16 | 0.081 | 0.252 |
|  | CaST | -0.19 | 0.061 | 0.252 |
|  | JLO | 0.08 | 0.426 | 0.532 |
|  | ST | 0.10 | 0.309 | 0.463 |
| **Intermediate** |  |  |  |  |
|  | ADL | 0.15 | 0.063 | 0.104 |
|  | FAQ | 0.10 | 0.192 | 0.241 |
|  | MoCA-B | -0.20 | 0.011 | 0.034 |
|  | AVLT long delay recall | -0.31 | 0.000 | 0.001 |
|  | AVLT cue recall | -0.24 | 0.003 | 0.011 |
|  | AVLT recognition | -0.20 | 0.015 | 0.037 |
|  | STT-A | 0.06 | 0.446 | 0.446 |
|  | STT-B | 0.15 | 0.070 | 0.104 |
|  | BVMT long delay recall | -0.33 | 0.000 | 0.001 |
|  | BVMT recognition | -0.26 | 0.001 | 0.007 |
|  | BNT | -0.08 | 0.316 | 0.339 |
|  | AFT | -0.17 | 0.025 | 0.054 |
|  | CaST | -0.16 | 0.063 | 0.104 |
|  | JLO | -0.09 | 0.292 | 0.337 |
|  | ST | -0.11 | 0.193 | 0.241 |
| **Late** |  |  |  |  |
|  | ADL | 0.18 | 0.023 | 0.045 |
|  | FAQ | 0.25 | 0.002 | 0.005 |
|  | MoCA-B | -0.15 | 0.063 | 0.086 |
|  | AVLT long delay recall | -0.35 | 0.000 | 0.003 |
|  | AVLT cue recall | -0.30 | 0.003 | 0.007 |
|  | AVLT recognition | -0.17 | 0.091 | 0.114 |
|  | STT-A | 0.23 | 0.024 | 0.045 |
|  | STT-B | 0.21 | 0.042 | 0.063 |
|  | BVMT long delay recall | -0.33 | 0.001 | 0.005 |
|  | BVMT recognition | -0.34 | 0.001 | 0.005 |
|  | BNT | -0.04 | 0.730 | 0.730 |
|  | AFT | -0.17 | 0.032 | 0.053 |
|  | CaST | -0.48 | 0.000 | < .001 |
|  | JLO | -0.04 | 0.690 | 0.730 |
|  | ST | -0.05 | 0.652 | 0.730 |

Note: Corrected for age, sex, and years of education. ADL, Activity of Daily Living; FAQ, Functional Activities Questionnaire; MoCA-B, Montreal Cognitive Assessment-basic; AVLT, Auditory Verbal Learning Test; BVMT, Brief Visuospatial Memory Test; AFT, Animal-Verbal Fluency Test; BNT, Boston Naming Test; STT, Shape Trail Test; CaST, Category Switching Test; JOL, Judgement of Line Orientation. ST, Silhouettes Test.

**Supplementary Table 14 Differences in the distribution of the two quaternary systems**

|  | **Count in Mattsson stage** | | | | | |  |  |
| --- | --- | --- | --- | --- | --- | --- | --- | --- |
| **Count in Grothe stage** | **Negative** | **Early** | **Intermediate** | **Late** | **UND** | Total | Statistic | P |
| **stage 0** | 514 | 0 | 0 | 0 | 0 | 514 | 3429.74 | <.001 |
| **stage 1+2** | 0 | 117 | 50 | 0 | 0 | 167 |  |  |
| **stage 3** | 0 | 0 | 117 | 6 | 0 | 123 |  |  |
| **stage 4** | 0 | 0 | 0 | 163 | 0 | 163 |  |  |
| **UND** | 0 | 0 | 0 | 0 | 35 | 35 |  |  |
| Total | 514 | 117 | 167 | 169 | 35 | 1002 |  |  |

Note: UND indicates "undetermined".

**Supplementary Table 15 Cognitive function and biomarker comparison in Grothe stage 1+2 and Grothe stage 3 in Mattsson early stage**

|  | Grothe stage 1+2 | Grothe stage 3 | Statistic | P | Effect Size |
| --- | --- | --- | --- | --- | --- |
| MoCA-B | 24.5(20.25, 27) | 22(17, 25) | 2358 | 0.047 | 0.19 |
| AVLT long delay recall | 5(3, 6) | 2(0, 5) | 1388 | < .001 | 0.42 |
| AVLT recognition | 22(20, 23) | 19(17, 22) | 1381.5 | < .001 | 0.42 |
| AFT | 14(11.25, 18) | 15(11, 17) | 2852 | 0.799 | 0.02 |
| BNT | 24.5(22, 26) | 23(20, 26) | 1976 | 0.103 | 0.17 |
| STT-A | 51(38, 62.25) | 51(42.75, 67) | 2055.5 | 0.193 | 0.13 |
| STT-B | 135(103.25, 171.75) | 144(117.5, 183.5) | 1805 | 0.062 | 0.2 |
| CaST | 15.5(13, 17) | 13(9.25, 15.75) | 1499 | 0.006 | 0.29 |
| JLO | 20(18, 23.25) | 20(17, 24) | 2178 | 0.711 | 0.04 |
| ST | 9(7, 12) | 9(7, 11) | 1875.5 | 0.144 | 0.15 |
| PTau 181 (pg/ml) | 1.78(1.39, 2.71) | 2.01(1.4, 2.6) | 1079 | 0.603 | 0.06 |
| Aβ 42/Aβ 40 | 0.06(0.04, 0.07) | 0.05(0.04, 0.06) | 996 | 0.264 | 0.14 |
| Nfl (pg/ml) | 13.44(10.78, 19.61) | 15.69(11.07, 20.95) | 977 | 0.21 | 0.15 |
| Left-Whole_hippocampal_head | 1617.73(1504.06, 1764.44) | 1482.88(1334.82, 1666.24) | 870 | 0.005 | 0.34 |
| Left-Whole_hippocampal_body | 1123.17(1087.02, 1181.83) | 1053.18(921.57, 1161.54) | 905 | 0.009 | 0.31 |
| Left-Hippocampal_tail | 539.13(473.02, 574.93) | 511.22(437.06, 561.73) | 1050 | 0.093 | 0.2 |
| Right-Whole_hippocampal_hea | 1665.47(1523.6, 1807.42) | 1605.24(1377.13, 1703.87) | 959 | 0.023 | 0.27 |
| Right-Whole_hippocampal_body | 1139.06(1077.65, 1225.39) | 1108.04(941.78, 1183.62) | 1004 | 0.048 | 0.24 |
| Right-Hippocampal_tail | 576.23(519.27, 631.71) | 543.97(476.52, 608.48) | 985 | 0.036 | 0.25 |

Note: The volume of the hippocampal subfields is measured in mm^3^. MoCA-B, Montreal Cognitive Assessment-basic; AVLT, Auditory Verbal Learning Test; AFT, Animal-Animal-Verbal Fluency Test; BNT, Boston Naming Test; STT, Shape Trail Test; SCWT, Stroop Word-Color Interference Task; CaST, Category Switching Test; JOL, Judgement of Line Orientation; ST, Silhouettes Test.
